# Time-series transcriptome provides insights into the gene regulation network involved in the icariin-flavonoid metabolism during the leaf development of *Epimedium pubescens*

**DOI:** 10.3389/fpls.2023.1183481

**Published:** 2023-06-12

**Authors:** Chaoqun Xu, Xiang Liu, Guoan Shen, Xuelan Fan, Yue Zhang, Chao Sun, Fengmei Suo, Baolin Guo

**Affiliations:** Key Laboratory of Bioactive Substances and Resources Utilization of Chinese Herbal Medicines, Ministry of Education & National Engineering Laboratory for Breeding of Endangered Medicinal Materials, Institute of Medicinal Plant Development, Peking Union Medical College and Chinese Academy of Medical Sciences, Beijing, China

**Keywords:** *Epimedium pubescens*, prenylated flavonol glycosides, time-series transcriptome, gene regulatory network, transcription factor

## Abstract

Herba Epimedii (Epimedium) leaves are rich in prenylated flavonol glycosides (PFGs) with high medicinal value. However, the dynamics and regulatory network of PFG biosynthesis remain largely unclear. Here, we combined metabolite profiling (targeted to PFGs) and a high-temporal-resolution transcriptome to elucidate PFGs’ regulatory network in *Epimedium pubescens* and identified key candidate structural genes and transcription factors (TFs) involved in PFG accumulation. Chemical profile analysis revealed that PFG content was quite different between buds and leaves and displayed a continuous decline with leaf development. The structural genes are the determinant reasons, and they are strictly regulated by TFs under temporal cues. We further constructed seven time-ordered gene co-expression networks (TO-GCNs) of PFG biosynthesis genes (including *EpPAL2*, *EpC4H*, *EpCHS2*, *EpCHI2*, *EpF3H*, *EpFLS3*, and *EpPT8*), and three flavonol biosynthesis routines were then predicted. The TFs involved in TO-GCNs were further confirmed by WGCNA analysis. Fourteen hub genes, comprising 5 MYBs, 1 bHLH, 1 WD40, 2 bZIPs, 1 BES1, 1 C2H2, 1 Trihelix, 1 HD-ZIP, and 1 GATA were identified as candidate key TFs. The results were further validated by TF binding site (TFBS) analysis and qRT-PCR. Overall, these findings provide valuable information for understanding the molecular regulatory mechanism of PFGs biosynthesis, enriching the gene resources, which will guide further research on PFG accumulation in *Epimedium*.

## Introduction

*Epimedium* herb (yin-yang-huo), a well-known traditional Chinese medicine (TCM), is recognized as a prominent prenyl-flavonol glycoside producer with high medicinal value. To date, only *Epimedium* and *Vancouveria* (sister genus of *Epimedium*, distributed in North America) have a high content of these prenylated flavonol glycosides (PFGs). Pharmacological evidence suggested that PFGs are the major active ingredients, and four of these (Epimedin A, Epimedin B, Epimedin C, and icariin) are used as important bioactive markers for quality control (Xie et al., 2010; Ma et al., 2011). PFGs possess superior capability in being neuro- and cardio-protective (Wang et al., 2007) and have also been used for enhancing reproductive function and anti-aging (Kang et al., 2012). In 2022, icaritin, an aglycone of all PFGs, was approved as a new drug to inhibit hepatocellular carcinoma (HCC) initiation and malignant growth (Zhu et al., 2011; Zhao et al., 2015). *Epimedium* species was predicted as an important and promising medicinal plant with broad market demand, but wild resources of medicinal *Epimedium* species have declined dramatically in recent years due to over-harvesting and habitat destruction (Zhang et al., 2009).

*Epimedium* is a leafy herbal medicinal plant. The information regarding PFG accumulation dynamics and regulation is scarce. The total PFG content, especially at harvest, is usually what is referred to in traditional use. A systematic understanding of PFG content dynamics could explore the genetic mechanisms and guide the harvesting practice. Huang et al. (2015) reported a study on the dynamic changes of PFGs in leaf developmental process of *E. sagittatum* and suggested that the total content (sum of the content of Epimedin A, B, C and icariin) peaked at folded young leaf with erected petiole stage and then sequentially decreased. Epimedin C constituted the main component and showed a similar trend.

To date, the majority of structural genes of PFGs biosynthesis pathway have been identified in *Epimedium* (Zeng et al., 2013a; Zeng et al., 2013b; Huang et al., 2015; Pan et al., 2017; Feng et al., 2018; Feng et al., 2019; Lyu, 2020; Yang X et al., 2020; Liu Y et al., 2021; Wang et al., 2021; Shen et al., 2022). Of these, four flavonoid skeleton genes, *EwPAL*, *Ew4CL1*, *EwCHS1*, and *EwCHI1* (Liu Y et al., 2021), one *EpsFLS* gene (Pan, unpublished), modification enzyme genes including two PTs, *EsPT2*, and *EpPT8* (Wang et al., 2021; Shen et al., 2022), prenylated flavonoid glycosides with a kind of glycosylation at the 7-OH position of the A-ring, *EpsGT8*, *EsGT1*,and *Ep7GT* (Feng et al., 2019; Yang X et al., 2020; Yao et al., 2022a), glycosylation at the 3-OH position of the C-ring, *EpsGT8*, *EsGT1*,*Ep7GT*, *Ek3RT*, and *Eps3RT* (Feng et al., 2018; Lyu, 2020), glycosylation at the 3-*O*-rhamnoside position, *EpF3R2″XylT* (Yao et al., 2022b), and one OMT gene, *EkOMT1* (Zhou J, 2021), have been functionally verified. The available public gene resources can be of great help in exploring the regulation of PFGs biosynthesis.

There have been few studies on TFs that focused on the regulation of the structural genes of flavonoids in *Epimedium*. Some TFs acted in a manner of MYB-bHLH-WD40 (MBW) complex. *EsAN2* (Huang et al., 2016b) and *EsMYBA1* (Huang et al., 2013) were reported to be involved in anthocyanin biosynthesis pathways and significantly enhanced the anthocyanin accumulation. *EsAN2* can significantly upregulate the expression of *CHS*, *CHI*, and *ANS*, while *EsMYBA1* regulated *CHS*, *CHI*, *F3H*, *DFR*, and *ANS*. In addition, *EsMYB7* and *EsMYB10* (Huang et al., 2012) were reported to regulate the PA biosynthesis, *EsTT8* or *EsGL3* (bHLH) and *EsTTG1* (WD40) may be the co-factors (Huang et al., 2015). Another type worked only by MYB, such as *EsMYBF1* (highly homologous with SG7), and positively regulated flavonol accumulation in a leaf-specific manner by strongly activating the expression of *EsF3H* and *EsFLS* (Huang et al., 2016a). *EsMYB12* and *EsMYB1*, belonging to SG4, have been implicated as transcriptional repressors and negatively regulated anthocyanin biosynthesis in all tissues and the biosynthesis of flavonoids in root, respectively (Huang et al., 2012). However, whether more TFs were involved in transcriptional regulation network remain unknown.

Chang et al. (2019) predicted a regulatory cascade of Kranz anatomy development, which is a structure crucial for the high efficiency of photosynthesis in C4 plants by establishing a time-ordered gene co-expression network (TO-GCNs) method that could use 3D (gene expression, condition, and time) time-series transcriptome data. The time order of TF genes in each gene co-expression network (GCN) was assigned by the breadth-first search algorithm initiated from a seed node which is monotonically increased or decreased. TO-GCNs can effectively elucidate relationships among TF *vs* TF and TF *vs* key genes during continuous development stages. Based on TO-GCNs, the potential regulators and cascade regulatory networks related to flower coloring of *Rhododendron simsii* and *Syringa oblata* were predicted (Yang F. et al., 2020; Ma et al., 2022), the UVB- and UVC-induced early physiological stress responses and the molecular mechanism were characterized in *Pinus tabuliformis* (Xu et al., 2021; Xu et al., 2022), and recently, poplar ‘84 K’ to salt treatment at time series was analyzed, and the physiological dynamics and the potential regulatory mechanism were solved (Zhao et al., 2023). Therefore, the application of time-series transcriptome can provide a new insight into the gene regulation network involved in PFGs.

Here, we report a comprehensive high-temporal-resolution investigation of transcriptome and metabolome (targeted to PFGs) of leaves at six development stages in *E. pubescens*. This study highlighted the regulatory mechanism underlying PFGs biosynthesis. We constructed seven TO-GCNs of TFs regulating structural genes in PFG pathways, and a regulation mechanism model was finally proposed. This study provides a road map for understanding the molecular regulatory mechanism of PFGs biosynthesis, which will facilitate further research on PFGs accumulation in *Epimedium*.

## Materials and methods

### Plant materials

Plant material *E. pubescens* was obtained from cultivation bases, Leshan, Sichuan province (43°50′9.66″N, 81°10′21.73″E), during spring to autumn (from 26 March to 26 August) of 2021. Analysis of the PFG content of the mature leaves of seven plants was conducted (three biological replicates for each plant, each repetition has six leaves) prior to the experiment, and three individuals with the closest content were used. The leaf width was preliminarily used as the criterion for determining different developmental stages. Leaf width of 0.5 **±** 0.2 ~ 5 **±** 0.2 cm with increments of 0.5 cm were collected. Samples were collected at 10:00~11:30 am of a sunny day and were respectively categorized into two parts used for PFG extraction and transcriptome. Each was treated with liquid nitrogen immediately after grafting, stored with dry ice, and quickly transported to Beijing for -80°C conservation under ultralow temperature and further used for RNA extraction and chemical component identification. In total, 42 samples were collected, including a terminal bud as well as 13 leaf sampling points, each with three replicates. Thirty-nine samples were finally used for transcriptome and metabolome determination (leaf width of 4.5 cm was not employed). Due to requirements of sequencing library construction and amount of extraction, 6 and 7 samples are missed, respectively. Finally, 33 and 32 samples were used, respectively, for transcriptome and metabolome analysis. Based on the PCA results of expression levels and PFG contents, the developmental stages were divided, and the sample numbers for each stage were reassigned, as detailed in **Supplemental Table 1** and **Supplemental Table 2**.

### PFGs identification and quantification

PFGs were extracted with 99.8% methanol and detected by ultra-high-performance liquid chromatography (UHPLC). Briefly, approximately 0.1 g of each sample was extracted using 1 ml of extraction solution by vortexing at 4°C and subsequent sonification in ultrasonic bath (RK100, Bandelin, Berlin, Germany) for 20 min, then the samples were centrifuged at 12,000 rpm for 10 min, and the supernatants were filtered through a 0.22 μm membrane. Chromatographic separations of compounds in methanol extracts were performed using a Waters ACQUITY I-Class UHPLC system coupled with photo-diode array and quadrupole time-of-flight mass spectrometry (UHPLC-PDA-Q-TOF/MSE) (Waters, Manchester, the United Kingdom). UHPLC-Q-TOF/MSE combined with the UNIFI data analysis platform were adopted to identify the PFGs. UHPLC-PDA was used to determine the relative content of PFGs. Chromatographic settings were as follows: the separation medium was performed on a Waters ACQUITY™ HSS T3 C18 column (100 mm × 2.1 mm) with 1.8 μm particle size (Waters, Ireland) at 40°C. The binary gradient elution system consisted of 0.1% formic acid-water (A) and acetonitrile (B) with a flow rate of 0.6 mL/min, and the absorbance was monitored at 270 nm. Separation was achieved using the following gradient: 0~1.5 min (21% B), 1.5~3 min (24% B), 3~4 min (25% B), 4~6.5 min (29% B), 6.5~7 min (32% B), 7~8 min (44% B), 8~9 min (45% B), 9~11 min (46% B) and 11~20 min (95% B). The injection volume was set to 2 μL. The mass spectrometer (MS) conditions were as follows: electronic impact ion source temperature, 110°C; auxiliary gas (N_2_) flow rate and temperature, 850 L/h and 450°C, respectively; negative and positive ionization mode were operated, and the capillary voltage was 2.5 and 3 kV, respectively; high and low scanning energy was 30-50 and 4 eV, respectively; the taper hole voltage, 50 V; the scanning range of molecular weight, 100-1,600 Da; and leucineenk ephalin solution was used to correct the accurate mass number.

Masslynx (version: 4.1) was used to analyze the chromatograms and mass spectra. Target compounds were identified by referring to Zhou M. et al. (2021). Peak area was utilized for quantification of all the target compounds. The PFGs standards used were homemade, including Hexandraside F, Epimedin A, Epimedin B, Epimedin C, icariin, 3’’’-carbonyl-2’’-β-*L*-quinovosyl-icariin, Ikarisoside B, 2’’-*O*-rhamnopyranosyl Ikarisoside A, Ikarisoside A, Sagittatoside A, Sagittatoside B, icariside I, 2’’-*O*-rhamnopyranosyl icariside II, icariside II and icaritin. The purity is more than 98%. Three independent experiments were performed, and the mean value was used for further analysis. Principal component analysis (PCA) was provided by R package factoextra. Log transformed and normalized PFGs were used as the inputs. Detailed scripts can be seen in **Supplemental File 1**.

### RNA exaction, library construction, and sequencing

Total RNA was extracted using TRIZOL reagent (Invitrogen, Life Technologies, USA) according to the manufacture’s protocol. NanoDrop ND 1000 (Nanodrop technologies) was initially used to detect the protein contamination, the ratio of OD260/OD280 was strictly controlled at 1.9~2.1, and then the RNA Integrity Number (RIN) was assessed by Agilent Technologies 2100 bioanalyzer (Agilent, Santa Clara,CA). Only when RIN > 8 and 28S/18S ≥ 0.7 was sequencing performed. 39 sequence libraries were constructed and sequenced on Illumina HiSeq 2500 platform in BioMED (https://www.biomedi.com.cn/).

### Transcriptome analysis

Trimmomatic (version: 0.36) was utilized to make quality control, raw reads were trimmed *via* removing adapters, low quality sequences or bases, and contaminations or overrepresented sequences. The clean data were mapped to the *E. pubescens* genome (Shen et al., 2022) by using HISAT2 (Kim et al., 2015), and hisat2-build and hisat2 were employed to build the index and make alignments, respectively. R package Rsubread (Liao et al., 2019) was adopted to perform gene expression quantification. Gene expression levels were calculated and normalized to transcripts per million (TPM) reads. Differentially expressed genes (DEGs) between each stage were identified with DESeq2 (Love et al., 2014). Genes with Benjamini-Yekutieli false discovery rate (FDR) < 0.05 and |log_2_(fold change)| >1 were considered to be DEGs. DEGs were subjected to enrichment analysis through gene ontology (GO) and Kyoto Encyclopedia of Genes and Genomes (KEGG) by using the R package clusterProfiler (version: 3.6.0) (Yu G et al., 2012). PCA was provided by R package PCAtools (Blighe and Lun, 2019). Log transformed and normalized gene expression data was used as the inputs. Detailed scripts can be seen in **Supplemental File 1**.

### TF prediction and PFG biosynthetic candidate gene identification

PlantTFDB database (http://planttfdb.gao-lab.org/index.php) and iTAK software (Zheng et al., 2016) were utilized for TF identification. PlantRegMap database (http://plantregmap.gao-lab.org/) was used to identify the WD40 family by homology to *Arabidopsis thaliana*. PFGs biosynthetic candidate genes were retrieved by using blast or the Hidden Markov Model (HMM) method embedded in HMMER (version: 3.0) (http://hmmer.org/). ClustalW2 (Larkin et al., 2007) and IQ-TREE (Minh et al., 2020) were used for sequence alignment and phylogenetic tree construction, and trees were visualized and modified using iTOL (https://itol.embl.de/) (Letunic and Bork, 2019).

### TO-GCNs construction

Three major steps were included for TO-GCNs (Chang et al., 2019): 1) Co-expression cutoffs determination, 2) gene co-expression network (GCN) construction, 3) Time-order of TF gene expression determination. TO-GCNs inputs were the expression profile for each expressed gene (with average TPM > 0.5), which consists of four time points (S1~S4). Pearson correlation coefficient (PCC) values for TFs and gene pairs were calculated, and the cutoff of positive co-expression PCC ≥ 0.85 (*p* < 0.01) was determined. TFs amounting to 1,020 and genes amounting to 20,966 above PCC ≥ 0.85 in “C1 + C2 +” GCN were constructed. MFSelector (Wong et al., 2020) was applied to identify the seed genes with ascending or descending monotonic patterns. A TF gene *FAR1* (*Ebr04G048560*) with the strongest ascending monotonic pattern was selected as the initial node to generate all time-ordered levels of nodes in the TO-GCNs by breath-first search algorithm. TO-GCNs were visualized by Cytoscape (version: 3.6.1) (Shannon et al., 2003). Detailed scripts can be seen in **Supplemental File 1**.

### Candidate PFGs genes regulatory network inference and TFBS analysis

TO-GCNs was firstly used to predict the candidate direct regulators, which should be co-expressed at the same level as or at one level earlier than the structural gene. Similarly, the second-, third-, and fourth-order candidate TFs were inferred, respectively. Secondly, TFBS analysis of each regulatory network of PFGs biosynthesis genes were predicted by extracting the 5’ upstream 2 Kb sequences and queried against PlantRegMap database. Thirdly, the presence of the TFBS in the promoter region of each network node were further checked.

### WGCNA analysis

All expressed genes (with average TPM > 0.5) were applied. Four major steps were included: 1) Co-expression modules were constructed by using the automatic network construction function blockwiseModules with parameters “soft thresholding power = 9”, “mergeCutHeight = 0.25”, and “minModuleSize = 50”; 2) An adjacency matrix and a subsequently topological overlap matrix (TOM) were constructed and converted, respectively; 3) Eigengene for each module was calculated and was used to correlate to PFG content. The networks were visualized by Cytoscape (version: 3.6.1) (Shannon et al., 2003). Detailed scripts can be seen in **Supplemental File 1**.

### qRT-PCR analysis

The pre-extracted RNA was reverse transcribed into cDNA using a HiScript II Reverse Transcriptase-based two-step qPCR kit (Vazyme Biotech Co. Ltd., Nanjing, China). Nine gene pairs were selected for validation. beta-Actin-1 was selected as the internal reference gene. Primer 5.0 software was used for primer design. The amplification system was constructed using a LineGene 9600 Plus quantitative real-time PCR detection system (Bioer, Hangzhou, China) and placed in CFX Connect (Bio-Rad Laboratories Inc. Hercules, CA, USA). Three technical replicates were used for each gene, and three biological replicates were used for samples of each developmental stage. The relative expression of genes was calculated using the 2^−ΔΔCt^ method. Origin (version: 2019) was used for correlation analysis to verify the credibility of transcriptome.

## Results

### Characterization and identification of PFGs in leaves of *E. pubescens*

The PFGs were identified as reported previously (Zhou M. et al., 2021). Here, a case for identifying Epimedin A **[** **Supplemental Figure 1**, the peak visible at retention time (R_t_) of 4.11 min] were illustrated. In high collision energy (CE) of ESI- mode, the peak eluting at R_t_ = 4.11 produced a fragment ion at *m/z* 367 [M+HCOO-2Glc-Rha]-, in combination with the evidence of a fragment ion at *m/z* 313 [M+H-2Glc-Rha-C_4_H_8_]+ in high CE of ESI+, the peak eluting at R_t_ = 4.11 was deduced to be a flavonoid of Type I **(** **Supplemental Figure 2** **)**. Then, based on the fragment ion at *m/z* 883 [M + HCOO]- in low CE of ESI- or at *m/z* 839 [M+H]+ in low CE of ESI+, the molecular mass was confirmed. Then the glycosyl chain at the C-3 site was inferred from the presence of *m/z* 839 [M+H]+ and *m/z* 677 [M+H-Glc]+ in low CE of ESI+, and a loss of 162 Da means the glycosyl ligand connected here is a glucose. The glycosyl chain at the C-7 site was evidenced by *m/z* 883 [M + HCOO]- and *m/z* 675 [M+HCOO-Glc]- in low CE of ESI-, and *m/z* 677 [M+H-glc]+ and *m/z* 531 [M+H-Glc-Rha]+ in low CE of ESI+, which suggested one glucose and rhamnose moiety, according to the database matching by UNIFI and literature reported (Zhao et al., 2008).Taken together, the peak eluting at R_t_ = 4.11 min was tentatively identified as Epimedin A (**Supplemental Figure 3C**). Detailed mass spectrogram of chromatographic for all identified PFGs can be seen in **Supplemental Figure 3**. Finally, a database was built based on the identified PFGs by UHPLC-Q-TOF/MS and PDA chemometric data, wherein only the commonly observed peaks in PDA chemometric data were used as the marker compounds. As a result, 14 types of PFGs were identified (**Table 1** and **Supplemental Figure 1**). Two backbone types of PFGs, belonging to anhydroicaritin (Type I, C-4′ linked methoxy) and demethylanhydroicaritin (Type II, C-4′ linked hydroxyl), were identified in all samplings **(** **Supplemental Figure 2** **)**, with ten and four PFGs included, respectively **(** **Table 1** **)**. Detailed major aglycone types, sugarmoieties, and substituent groups in leaves of *E. pubescens* are summarized in **Table 1**.

**Table 1:** UHPLC-Q-TOF/MS metabolic fingerprinting of methanol extracts of *E. pubescens* buds (S0) and leaves of five developmental stages (S1~S5).

Peak No.	Rt (min)	Compound	Molecular formula	Calculated mass (m/z)	Fragment ions (m/z)	Aglycone type	R1	R2	S0	S1	S2	S3	S4	S5
1	2.07	Diphyllodside B	C_38_H_48_O_19_	807.2701	645.2173, 353.1035 (-)	II	Rha-Rha	Glc	+	+	+	+	+	+
				809.2852	517.1718, 355.1573, 299.0548 (+)									
2	2.19	Epimedoside A	C_32_H_38_O_15_	661.2251	499.1683, 353.1010 (-)	II	Rha	Glc	+	+	+	+	+	+
				663.2525	517.1907, 355.1298 (+)									
3	4.11	Epimedin A	C_39_H_50_O_20_	837.294	675.2376, 367.1208 (-)	I	Rha(2-1)Glc	Glc	–	+	+	+	+	+
				839.3212	677.2615, 531.2004, 369.1444, 313.0804 (+)									
4	4.32	Epimedin B	C_38_H_48_O_19_	853.2689	645.2260, 367.1208 (-)	I	Rha-Xyl	Glc	–	+	+	+	+	+
				809.3088	677.2648, 531.2004, 369.1444, 313.0804 (+)									
5	4.52	Epimedin C	C_39_H_50_O_19_	867.3208	659.2418, 513.1812, 367.1208 (-)	I	Rha(2-1)Rha	Glc	+	+	+	+	+	+
				823.3237	677.2615, 531.2004, 369.1240, 313.0804 (+)									
6	4.71	Icariin	C_33_H_40_O_15_	721.2438	513.1832, 367.1208 (-)	I	Rha	Glc	+	+	+	+	+	+
				677.2681	531.2062, 369.1469, 313.0827 (+)									
7	5.5	3’’’-carbonyl-2’’-β-*L*-quinovosyl-icariin	C_39_H_48_O_19_	819.2817	657.2256, 513.1832, 367.1208(-)	I	Rha(2-1)Qui	Glc	–	+	+	+	+	+
				821.3079	531.2004, 369.1444, 313.0804 (+)									
8	6.15	Anhydroicaritin-3-*O*-(acetyl)rhamnopyranosyl-xylopyranosyl-7-*O*-glucopyranoside	C_40_H_50_O_20_	819.2817	657.2256, 513.1832, 367.1208(-)	I	Rha(OAc)Xyl	Glc	–	+	+	+	+	+
				821.3079	531.2004, 369.1444, 313.0804 (+)									
9	7.36	2’’-*O*-rhamnosyl-ikarisoside A	C_32_H_38_O_14_	645.2173	352.0936(-)	II	Rha-Rha	H	+	–	–	–	–	–
				647.2309	501.1755,355.1150 (+)									
10	7.42	Anhydroicaritin-3-*O*-(acetyl) rhamnopyranosyl-(acetyl)xylopyranosyl-7-*O*-glucopyranoside or its isomers	C_42_H_52_O_21_	937.3149	729.2535, 367.1233 (-)	I	Rha(OAc)-Xyl(OAc)	Glc	–	+	+	+	+	+
				893.3442	719.2871, 531.2092, 369.1493 (+)									
11	7.46	Anhydroicaritin-3-*O*-(acetyl) rhamnopyranosyl-(acetyl)xylopyranosyl-7-*O*-glucopyranoside or its isomers	C_42_H_52_O_21_	937.3149	729.2535, 367.1233 (-)	I	Rha(OAc)-Xyl(OAc)	Glc	–	+	+	+	+	+
				893.3442	719.2871, 531.2092, 369.1493 (+)									
12	7.53	Ikarisoside A	C_26_H_28_O_10_	499.1626	353.1018 (-)	II	Rha	H	+	–	–	–	–	–
13	8.26	2’’-*O*-rhamnopyranosyl icariside II	C_33_H_40_O_14_	659.2418	367.1208, 352.0966 (-)	I	Rha(2-1)Rha	H	+	+	+	+	+	+
				661.2669	515.2066, 369.1444, 313.0804 (+)									
14	8.77	Icariside II	C_27_H_30_O_10_	513.1832	366.1153, 351.0904, 323.0949 (-)	I	Rha	H	+	+	+	+	+	+
				515.2066	369.1444, 313.0804 (+)									

### Division of leaf development stages

**Figure 1:**
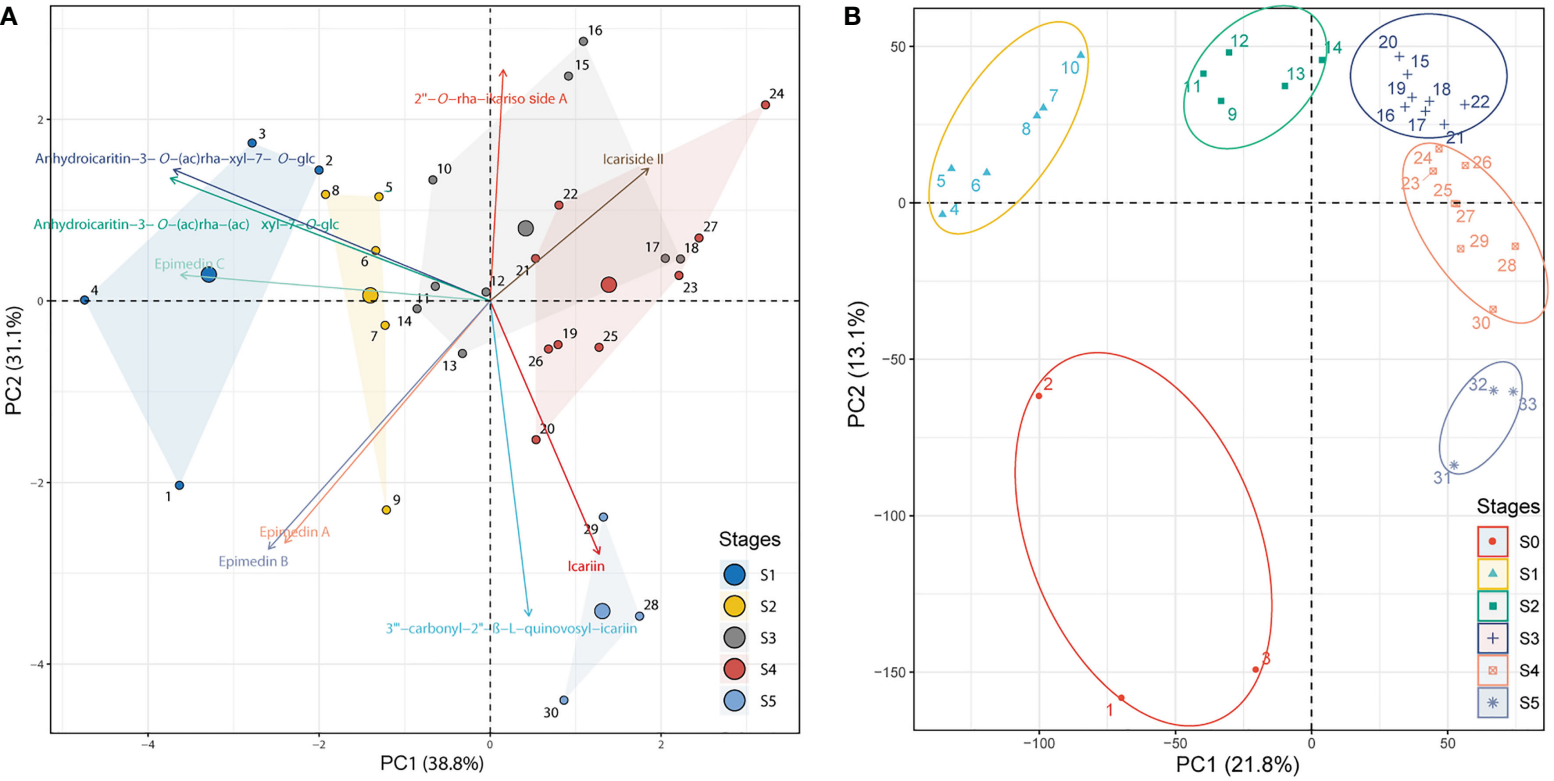
PCA of identified PFGs and gene expression levels. **(A)** PCA of the content of identified PFGs in all collected samples from S1~S5. The PCA biplot shows both the PC scores of zero-centered and unit-scaled compound quantity data (the dots represented the sampling individuals) and the loadings of variables (the vectors represented the identified PFGs). Seven individuals are not displayed because the insufficient samplings could not meet the extraction requirements. See **Supplemental Table 1** for the numerical symbols. **(B)** PCA of gene expression levels in S0~S5. All expressed genes (average TPM > 0.5) for all collected samples except for six ambiguous individuals were displayed. See **Supplemental Table 2** for the numerical symbols.

**Figure 2:**
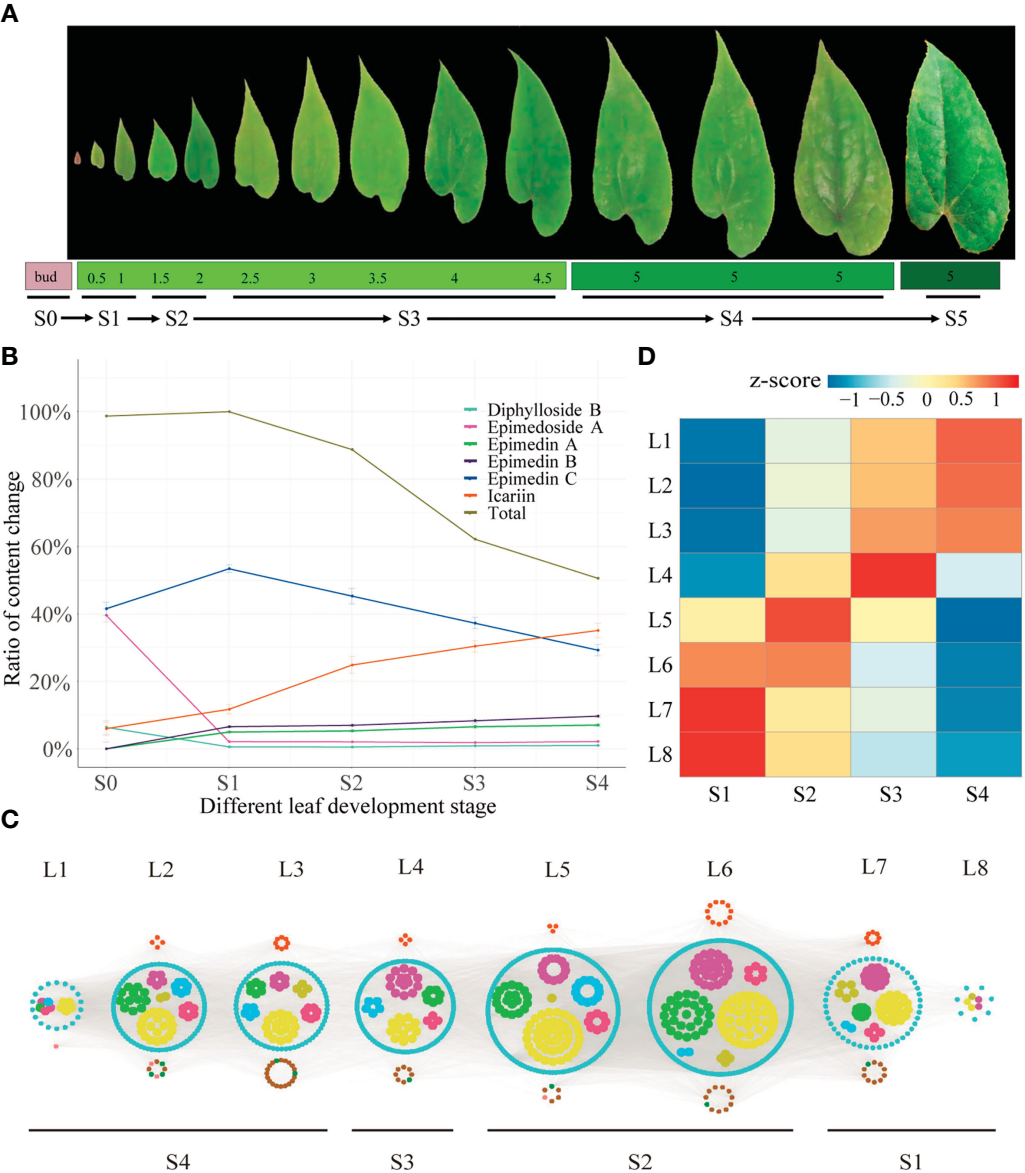
Time-ordered gene co-expression network related to leaf development in *E. pubescens*. **(A)** Schematic representation of the design for samplings of *E pubescens.* Leaf width was preliminarily taken as the division of leaf development stages, 42 samples from 14 sampling points, each with three replicates, were collected for metabolic profiling and RNA-seq. Six stages were defined: 1) Stage 0 (S0, bud stage), 2) Stage 1 (S1, leaf width of 0.5~1 cm), 3) Stage 2 (S2, leaf width of 1.5~2 cm), 4) Stage 3 (S3, leaf width of 2~4 cm), 5) Stage 4 (S4, leaf width of 5 cm with middle degree of leathery), and 6) Stage 5 (S5, leaf width of 5 cm with high degree of leathery); **(B)** Proportion of content for the main PFGs in S1~S5 of *E pubescens*. Gray line: total PFGs, blue line: Epimedin C, orange line: icariin, purple line: Epimedin B, green line: Epimedin A, cyan line: Diphylloside B, pink line: Epimedoside A; **(C)** Predicted regulatory network and the connection among TFs and the structural genes involved in PFGs biosynthesis pathway. Inside the cyan circles, purple nodes represented *MYB* genes, green nodes represented *bHLH* genes, yellow nodes represented WD40 genes, brown nodes represented *bZIP* genes, light-blue nodes represented *WRKY* genes, light-green nodes represented *ARF* genes. Outside cyan circles, red nodes, locating on the top, represented *PAL*, *C4H*, *4CL*, *CHS*, *CHI*, *F3H*, and *FLS* genes, and green, brown, and pink nodes on the bottom represented *PT*, *UGT*, and *OMT* genes, respectively; **(D)** The heatmaps of average normalized TPM (z-score) at S1~S4 stages at each level were identified in the time-ordered gene co-expression network. Four stages of leaf with different types of flavonoids accumulation were identified, S1 (L8 and L7), S2 (L6 and L5), S3 (L4), and S4 (L3, L2 and L1) based on the expression profile. The bar represents the expression level of each gene (z-score). Low to high expression is indicated by a change in color from blue to red.

The development stages were defined based on the integration of PCA analyses against the metabolome (targeted to PFGs) **(** **Supplemental Table 1** **)** and transcriptome **Supplemental Table 2** **)** data, respectively. PCA of metabolome data (**Figure 1A**) showed that the first two PCs cumulatively accounted for ~70% of the total variance. PC1 revealed a clear separation among samples of 1~4 (leaf width of 0.5~1 cm), 5~9 (leaf width of 1.5~2 cm), and 10~30 (leaf width of 2.5~5 cm). These groupings were arranged in a clear time-series manner (from left to right), but samples of 28~30 (old leaf with highly leathery) displayed a different characteristic with samples of 10~27 (leaf width of 2.5~4 cm with middle leathery) in PC2 (**Supplemental Table 1**). PCA of transcriptome data (expressed genes, defined as average TPM > 0.5) exhibited a similar trend **(** **Figure 1B** **)**. PC1 showed dynamic changes over the time-series (from left to right). Samples of 4~10 (leaf width of 0.5~1 cm) and 9~14 (leaf width of 1.5~2 cm) revealed significantly different characteristics and were distinguished from 15~33 (leaf width of 2.5~5 cm), which showed a different characteristic in PC2. Notably, samples of 1~3 (bud stage) was with significantly different characteristics **Supplemental Table 2**). To sum up, the leaf development can be segregated into six stages: 1) Stage 0 (S0), bud stage; 2) Stage 1 (S1), leaf width is 0.5~1 cm, with low-degree of leathery; 3) Stage 2 (S2), leaf width is 1.5~2 cm, with low-degree of leathery; 4) Stage 3 (S3), leaf width is 2~4 cm, with low-degree of leathery; 5) Stage 4 (S4), leaf width is 5 cm, with middle-degree of leathery; 6) Stage 5 (S5), leaf width is 5 cm, with high-degree of leathery **(** **Figure 2A** **)**.

### Dynamic changes of PFGs with leaf development

The dynamic changes of PFGs were further revealed. Firstly, bud stage (S0) showed the exclusivity of chemical composition and content accumulation. Ikarisoside A and Rhamnose-ikarisoside A were observed as marker components, but they were not detected at leaf stages (S1~S4), Epimedin A and B were almost non-existent in bud stage (S0), with a very low proportion in leaf stages and were almost unchanged (4.97~7.03% for Epimedin A, 6.56~9.69% for Epimedin B) with leaf development (S1~S4). In addition, Epimedoside A and Diphylloside B showed higher contents in bud stage (S0), which was much lower at leaf stages (~39.60% in S0 and ~2.0% in S1~S4 for Epimedoside A; ~6.37% in S0 and ~0.70% in S1~S4 for Diphylloside B). Secondly, leaf stages (S1~S4) showed similar performance in chemical compositions but with significant changes in content accumulation. Total PFGs content were almost unchanged from S0 to S1 (~1.30% increased) and peaked at S1 (the highest accumulation) and then decreased rapidly, followed by 11.23%, 37.81% and 49.43% decreases from S1 to S2, S1 to S3, and S1 to S4, respectively. Similarly, Epimedin C showed the largest proportion of S1 (~53.41% of the total PFGs) and with changes similar to total PFG content, with an ~11.87% increase from S0 to S1, followed by continuous decrease, with an ~8.11%, ~16.14%, and ~24.15% decrease from S1 to S2, S1 to S3, and S1 to S4, respectively. However, the content of icariin was gradually increased, with amplification of ~5.76%, ~13.11%, ~18.71%, and ~23.36% in S0 to S1, S1 to S2, S1 to S3, and S1 to S4, respectively **(** **Figure 2B**, **Table 1** and **Supplemental Table 3** **)**.

### Transcriptome profiles at different development stages of *E. pubescens*

We generated 5.62~9.04 Gb clean bases per library and a total of 923 Mb clean pair-end reads after filtering and removing the adapter sequences. Q20, Q30, and GC content were higher than 97, 93, and 44%, respectively **Supplemental Table 4** **)**. The clean reads were mapped to the *E. pubescens* reference genome with an average alignment rate of 87.90% **Supplemental Table 5** **)**, and 21,345 genes were found to be expressed in at least one sample **(** **Supplemental Table 2** **)**.

By comparing the overrepresented GO categories among the DEGs, the biological processes of each stage were outlined. Compared with S0, it was notable that S1 showed an upregulation of basic energy metabolism and antioxidant capacity **(** **Supplemental Figure 4** and **Supplemental Table 6** **)**. Compared with S1, the up-regulated genes at S2 mostly have function in processes relevant to “cell wall organization or biogenesis (GO:0071555, GO:0042546)”, indicating a shift to plant protection, and this change lasted until S3. Multiple enzyme-encoding genes, for example, chitinase, laccase, peroxidase and pectin esterase, were involved. Notably, the PFG content revealed a rapid decline between S2 and S3, and further analysis showed that ‘‘secondary metabolic process (GO:0019748)” was overrepresented in the S2 *vs* S3 up-regulated gene set. This GO term included 6 genes, and 3 genes (*Ebr03G037830*, *Ebr03G037820*, and *Ebr02G014470*) were Glutathione S-transferase (GST), which may affect the accumulation of PFG content **(** **Supplemental Table 6** **)**. Compared with S3, GO terms related to “abscisic acid-activated signaling pathway” emerged at up-regulated gene set of S4 *vs* S3, however, biological processes relevant to “cell wall organization or biogenesis”, “lignin catabolic process”, and “hormone-mediated signaling pathway”, especially “ethylene-mediated signaling pathway” were overrepresented in the down-regulated gene set of S4 *vs* S3 **(** **Supplemental Figure 4** and **Supplemental Table 6** **)**. This observation indicates that ABA and ethylene signal transduction tend to play a major role in regulating leaf development or functional transition. To test this hypothesis, we further explored the gene expression pattern from S1 to S4, which reflected the largest variation of PFG content. The results of GO **(** **Supplemental Figure 5** **)** and KEGG enrichment **(** **Supplemental Figure 6** **)** of up-regulated gene set of S1 *vs* S4 were in line with the above-mentioned studies **(** **Supplemental Table 6** **)**.

### Mining of PFGs biosynthetic genes and TFs

**Figure 3:**
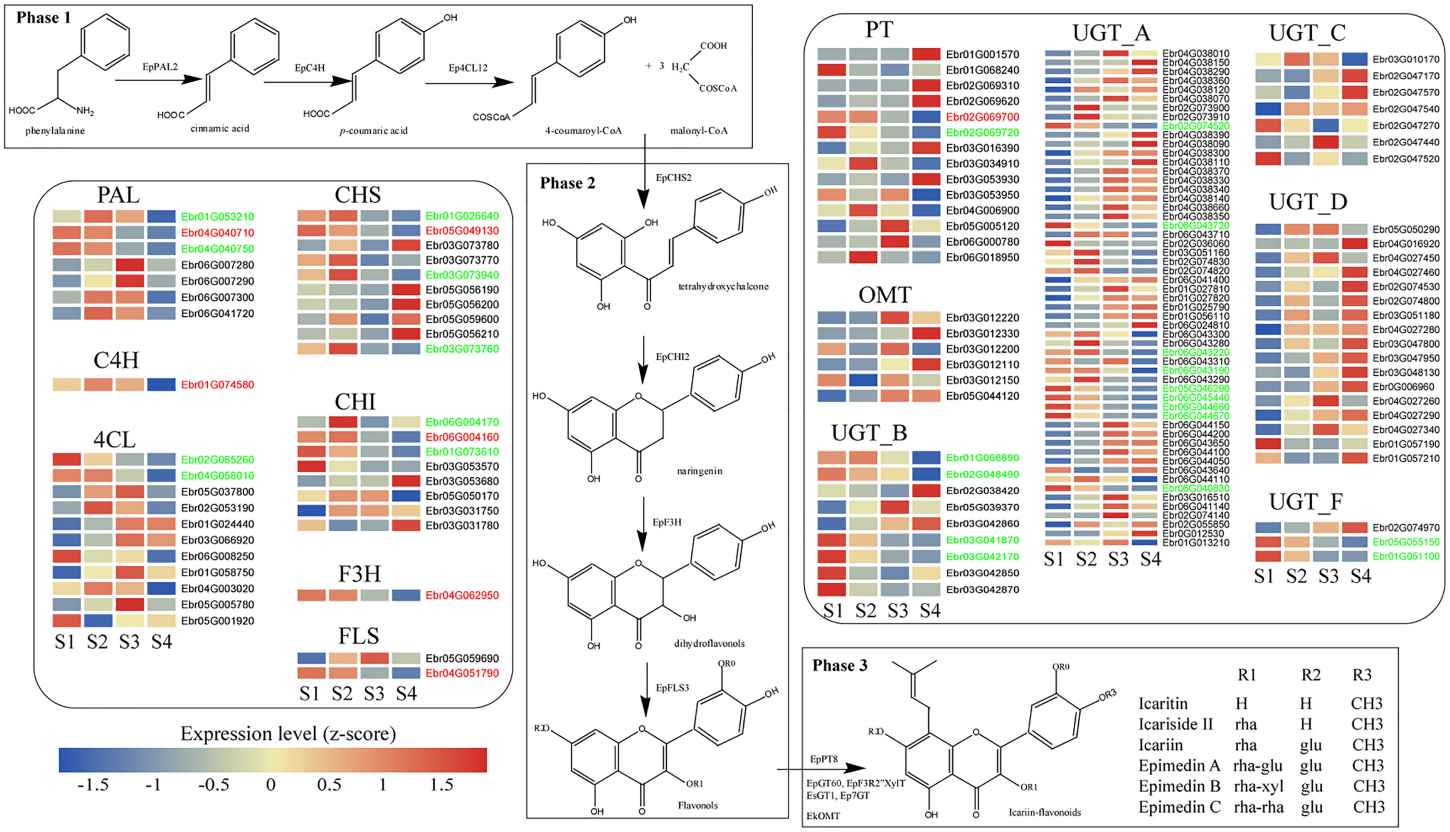
The biosynthesis pathway of PFGs in *E. pubescens*. The PFG biosynthesis pathway was divided into three phases. Phase 1: the common phenylpropanoid metabolism, including PAL: phenylalanine ammonia-lyase, C4H: cinnamate-4-hydroxylase, and 4CL: 4-coumarate CoA ligase; Phase 2: core pathway, including CHS: chalcone synthase, CHI: chalcone isomerase, F3H: flavanone 3-hydroxylase, and FLS: flavonol synthase; Phase 3: further enzymatic modification, including PT: prenyltransferase, UGT: UDP-glycosyltransferase, and OMT: *O*-methyltransferase; this study showed genes related to flavonoid biosynthesis (group A, B, C, D and F of UGT gene family, referring to Yao et al., 2022a). Gene expression profiles (in normalized TPMs) in different stages (here S1~S4, from left to right in each heatmap panel) are presented in the heatmap alongside the gene names. Gene names marked in red color represent genes with functional verification, genes marked in green color represent genes consistent with content change during leaf development. The bar represents the expression level of each gene (z-score). Low to high expression is indicated by a change in color from blue to red.

PFGs biosynthetic genes and TFs needed to be mined before constructing TO-GCNs. A total of 259 PFGs biosynthetic genes including 7 PALs (*EpPAL1~EpPAL7*) (Xu et al. unpublished), 1 C4H (*EpC4H*), 14 4CLs (*Ep4CL1~Ep4CL14*), 12 CHSs (*EpCHS1~EpCHS12*) (Shen et al. unpublished), 2 CHIs (*EpCHI1~EpCHI2*) (Fan et al. unpublished), 1 F3H (*EpF3H*), 3 FLSs (*EpFLS1~EpFLS3*), 19 PTs (*EpPT1~EpPT19*) (Shen et al., 2022), 183 UGTs (Yao et al., 2022a) and 17 OMTs (*EpOMT1~EpOMT17*) (Shen et al. unpublished) were identified. Through blast results with the activity verification reported genes **(** **Supplemental Table 7** **)**, the matching between expression level with PFGs content during leaf development and the mutation analysis of key sites (including substrate-binding site, active site or phosphorylation site) **Supplemental Table 8**), 9 genes [including *EpPAL2* (*Ebr04G040710*), *EpC4H* (*Ebr01G074580*), *Ep4CL2* (*Ebr04G003020*), *EpCHS2* (*Ebr05G049130*), *EpCHI2* (*Ebr06G004160*), *EpCHIL* (*Ebr01G073610*), *EpF3H* (*Ebr04G062950*), *EpFLS3* (*Ebr04G051790*) and *EpPT8* (*Ebr02G069700*)] were selected as the candidate genes that participated in the PFGs biosynthesis of *E. pubescens* (**Figure 3**, **Supplemental Figure 7**, **Supplemental Table 8**). A total of 2,249 TFs were detected in *E. pubescens* genome, which were classified into 59 families according to the PlantTFDB database **(** **Supplemental Table 9** **).** A total of 1,208 TF genes were expressed (average TPM > 0.5) in S1~S4. WD40, MYB, bHLH, ERF, and C2H2 families accounted for the largest portion, comprising 195, 97, 94, 80, and 52 members, respectively (**Supplemental Table 10**).

### TO-GCNs regulatory network construction

Between any two leaf developmental stages (S1~S4), 20,943 genes (1,208 TFs and 19,735 structural genes) were expressed (average TPM > 0.5). A TF gene *FAR1* (*Ebr04G048560*), expressing in a low level at S1 and monotonically increasing until S4, was selected as the initial node to build a TO-GCNs network. Eight time-series expression levels (L1~L8, nodes > 10) centering on TFs were finally constructed using the suggested positive/negative cutoff values (0.85; -0.61). Finally, 1,124 genes including 1,022 TFs and 102 PFGs biosynthesis genes made up the TO-GCNs specific to PFGs biosynthetic pathway **(** **Figure 2C** **).** These eight levels were corresponded to the average expression levels at the four developmental stages (S1~S4), as shown by the red squares (high expression levels) along the diagonal in the heatmap, which formed the basis for the inference of upstream and downstream genes/metabolites regulatory relationships **(** **Figure 2D** **)**.

With regard to the established TO-GCNs, the major PFGs biosynthetic genes were mainly expressed at the earlier stages (S1~S2) **(** **Supplemental Table 11** **)**. There were 18 and 31 PFGs biosynthetic genes expressed in S1 and S2, respectively. The co-expression genes of S1 and S2 reflected a higher expression in early time, and a lower expression in lateral time, and this was in line with PFGs content changes. *EpPT8* (*Ebr02G069700*), *EpPAL2* (*Ebr04G040710*), and *EpFLS3* (*Ebr04G051790*) were presented in L7. *Ep4CL12* (*Ebr04G003020*), *EpC4H* (*Ebr01G074580*), *EpCHS2* (*Ebr05G049130*), *EpCHI2* (*Ebr06G004160*), *EpCHIL* (*Ebr01G073610*), and *EpF3H* (*Ebr04G062950*) were found in L6. Very few causal genes of PFGs biosynthesis could be detected in L1~L4.

### Regulatory network prediction of PFG biosynthetic genes

**Figure 4:**
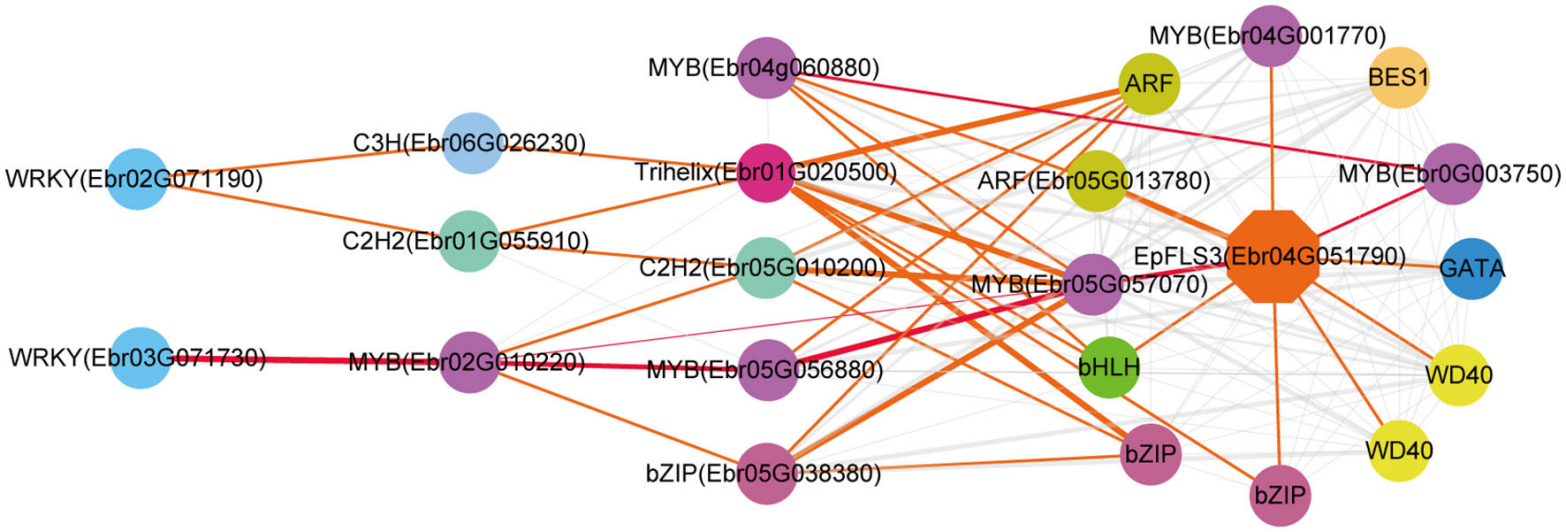
Resolved hierarchical regulation for *EpFLS3*. Thickness of the edges in any gene pair represent the strength of co-expression relationships. Thick red and orange edges mean a strong correlation (> 0.8), thin red and orange edges, a middle correlation (0.7~0.8), and gray edges, a weak correlation (0.66~0.7). Red edges represented the most probable pathway related to PFGs biosynthesis.

The regulatory network of seven candidate genes (*EpPAL2*, *EpC4H*, *EpCHS2*, *EpCHI2*, *EpF3H*, *EpFLS3*, and *EpPT8*) were predicted, and the TFBS results of each regulatory network are shown in **Supplemental Table 12**. Take *EpFLS3* for example **(** **Figure 4** **)**. We suggest that *EpFLS3* may be regulated in a hierarchical order by three routines: 1) *WRKY* (*Ebr03G071730*) acted as the fourth regulator. It directly regulated the third regulator *MYB* (*Ebr02G010220*), then regulated *MYB* (*Ebr05G056880*), then regulated *MYB* (*Ebr05G057070*), and finally regulated *EpFLS3*. 2) *WRKY* (*Ebr02G071190*) acted as the fourth regulator, either *C3H* (*Ebr06G026230*) or *C2H2* (*Ebr01G055910*) acted as the third regulators, both may regulate *Trihelix* (*Ebr01G020500*), which acted as second regulator and regulated *MYB* (*Ebr05G057070*), and *MYB* (*Ebr05G057070*) served as the direct regulator of *EpFLS3*; on the other hand, *C2H2* (*Ebr01G055910*) could also regulate *MYB* (*Ebr05G057070*) and then regulated *EpFLS3*. 3) *MYB* (*Ebr04G060880*) acted as the second regulator, regulated *MYB* (*Ebr0G003750*), then regulated *EpFLS3*. The routines of 1 and 3 were the most likely regulatory pathways, as *MYB* (*Ebr02G010220*) and *MYB* (*Ebr0G003750*) have been proven to participate in flavonol biosynthesis, which were homologous genes with *A. thaliana* MYB genes *AtMYB111*, *AtMYB11*, and *AtMYB12*. *MYB* (*Ebr02G010220*), *MYB* (*Ebr0G003750*), and *MYB* (*Ebr05G057070*) may act as the core regulated genes for *EpFLS3* regulation.

**Figure 5:**
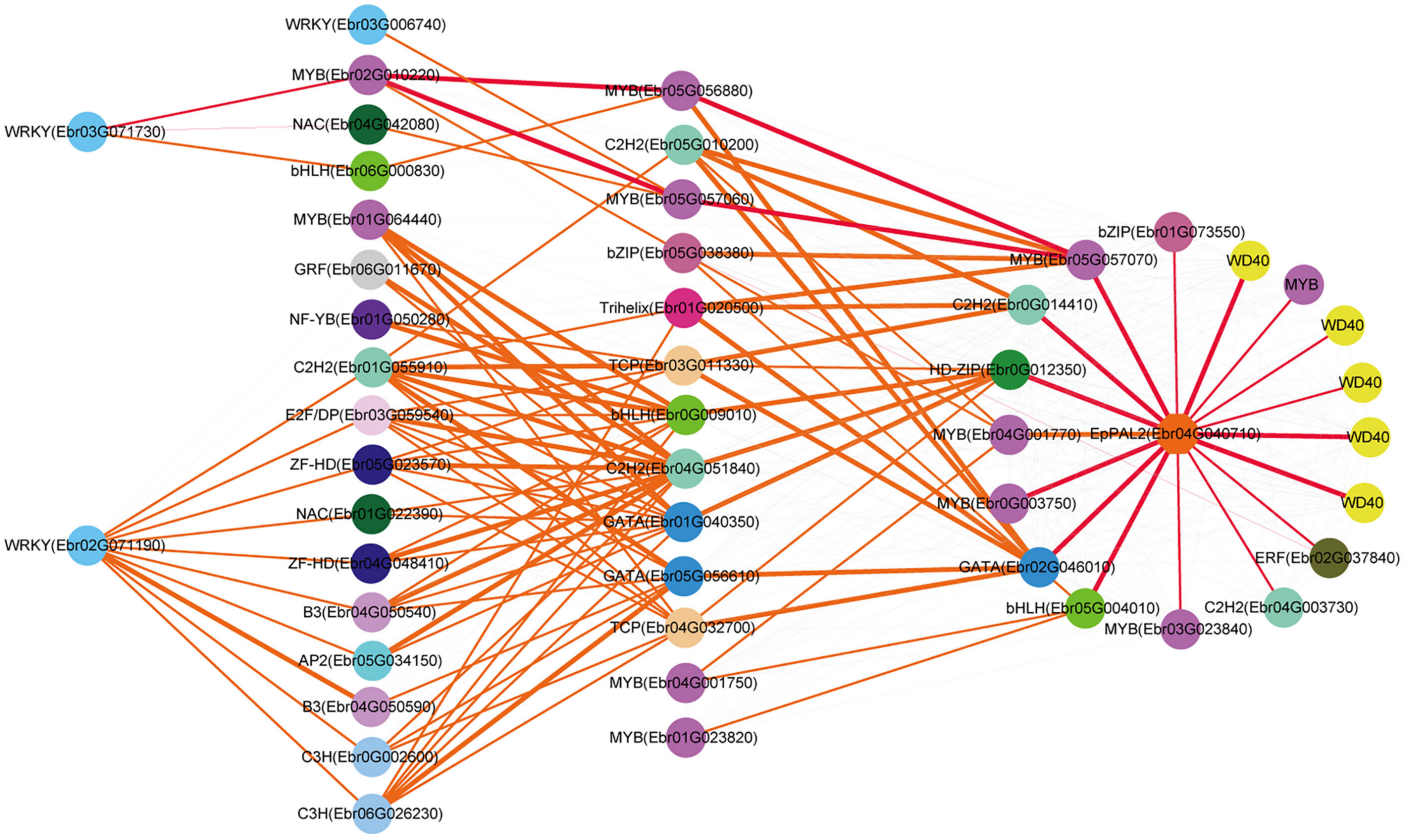
Resolved hierarchical regulation for *EpPAL2*. The notion is the same as **Figure 4**.

**Figure 6:**
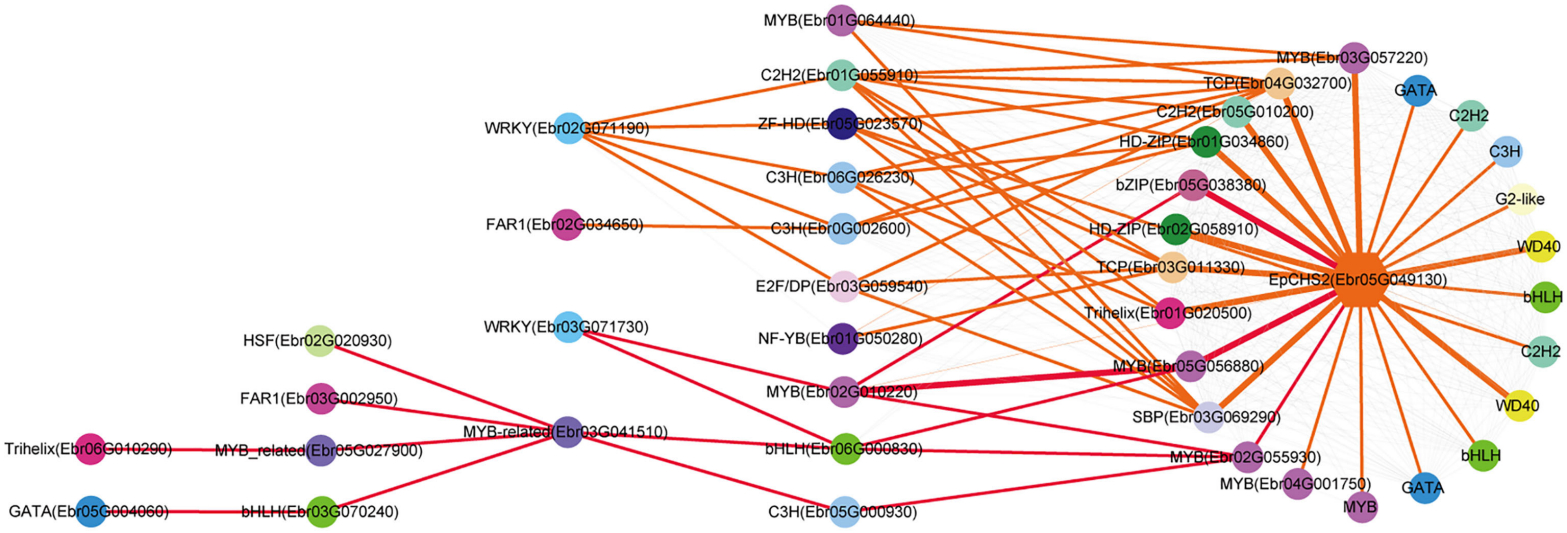
Resolved hierarchical regulation for *EpCHS2*. The notion is the same as **Figure 4**.

Both *EpPAL2* and *EpCHS2* showed more complex regulatory networks than other PFGs biosynthetic genes. In brief, the direct regulators of *EpPAL2* with correlation level over 0.8 were six genes except WD40. These genes [*MYB* (*Ebr05G057070*), *C2H2* (*Ebr0G014410*), *HD-ZIP* (*Ebr0G012350*), *MYB* (*Ebr0G003750*), *GATA* (*Ebr02G046010*), and *bHLH* (*Ebr05G004010*)] were further regulated by the second regulators with different correlation levels, and finally, the core regulatory networks of *EpPAL2* were predicted. 1) *WRKY* (*Ebr03G071730*) -> *MYB* (*Ebr02G010220*) -> *MYB* (*Ebr05G056880*) *-> MYB* (*Ebr05G057070*) *-> EpPAL2*. 2) *WRKY* (*Ebr03G071730*) -> *MYB* (*Ebr02G010220*) *-> MYB* (*Ebr05G057060*) *-> MYB* (*Ebr05G057070*) *-> EpPAL2* **(** **Figure 5** **)**. Similarly, the most probably regulatory routines of *EpCHS2* were as follows: 1) *WRKY* (*Ebr03G071730*) -> *MYB* (*Ebr02G010220*) *-> MYB* (*Ebr05G056880*) *-> EpCHS2*. 2) *WRKY* (*Ebr03G071730*) -> *MYB* (*Ebr02G010220*) *-> bZIP* (*Ebr05G038380*) *-> EpCHS2*. In addition, *WRKY* (*Ebr03G071730*) -> *MYB* (*Ebr02G010220*) *-> MYB* (*Ebr02G055930*) *-> EpCHS2*, *MYB-related* (*Ebr03G041510*) -> *C3H* (*Ebr05G000930*) -> *MYB* (*Ebr02G055930*) *-> EpCHS2*, *MYB-related* (*Ebr03G041510*) -> *bHLH* (*Ebr06G000830*) -> *MYB* (*Ebr02G055930*) *-> EpCHS2* may also be possible candidate routines, as *MYB* (*Ebr02G055930*) is involved in flavonol biosynthesis **(** **Figure 6** **)**. By further analysis of the regulatory network of *EpC4H* (**Supplemental Figure 8**), *EpCHI2* (**Supplemental Figure 9**), and *EpF3H* (**Supplemental Figure 10**), it was found that those genes harbored similar regulatory relationships to *EpPAL2*, *EpCHS2*, and *EpFLS3*. Our research suggests that a set or several sets of TFs, acting in a collaborative manner, regulated the biosynthesis pathway of PFGs.

**Figure 7:**
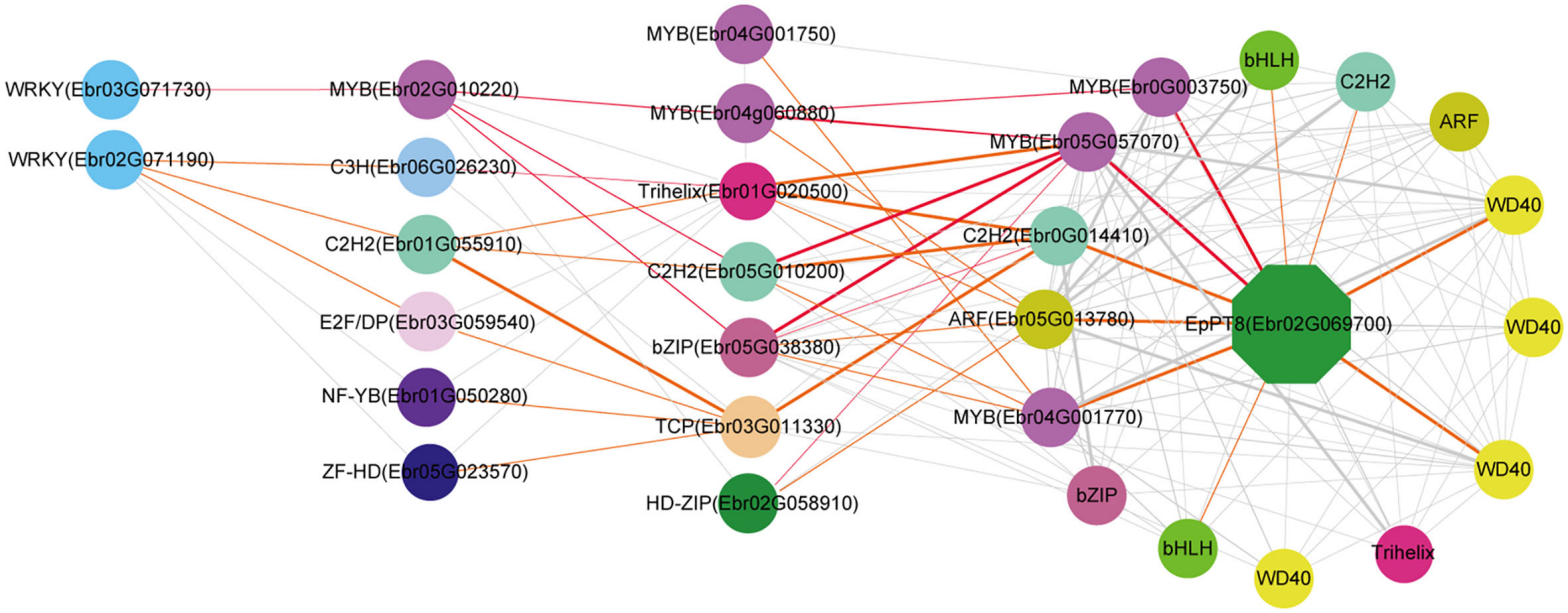
Resolved hierarchical regulation for *EpPT8*. The notion is the same as **Figure 4**.

We further predicted the regulatory network of *EpPT8*, which was the important gene for the formation of active ingredients of *Epimedium*. *MYB* (*Ebr05G057070*), *MYB* (*Ebr0G003750*), *C2H2* (*Ebr0G014410*), *ARF* (*Ebr05G013780*), and *MYB* (*Ebr04G001770*) were predicted to be the direct regulators. *Trihelix* (*Ebr01G020500*), *C2H2* (*Ebr05G010200*), *bZIP* (*Ebr05G038380*), and *TCP* (*Ebr03G011330*) acted as the secondary regulators. The predicted regulatory routines may be as follows: 1) *WRKY* (*Ebr03G071730*) -> *MYB* (*Ebr02G010220*) *-> C2H2* (*Ebr05G010200*) *-> MYB* (*Ebr05G057070*) *-> EpPT8*; 2) *WRKY* (*Ebr03G071730*) -> *MYB* (*Ebr02G010220*) *-> bZIP* (*Ebr05G038380*) *-> MYB* (*Ebr05G057070*) *-> EpPT8*; and 3) *WRKY* (*Ebr03G071730*) -> *MYB* (*Ebr02G010220*) *-> MYB* (*Ebr04G060880*) *-> MYB* (*Ebr0G003750*) *-> EpPT8* **(** **Figure 7** **).**

### Exploring TFs involved in PFG accumulation based on WGCNA analysis

**Figure 8:**
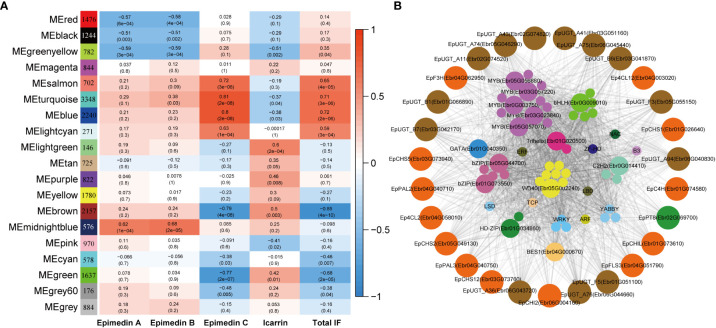
WGCNA of all expressed genes. **(A)** Module-PFGs relationship. Each row represented a module, which consists of genes with similar expression pattern. Each column represented the specific PFGs compound content, including Epimedin A, B, C, and icariin, and the sum of all PFGs. The value in each cell at the row-column intersection represents the correlation coefficient between the module and the specific compound content and is displayed according to the color scale on the right. The value in parentheses in each cell represents the *P* value. **(B)** Regulatory network of PFG biosynthesis in *E pubescen*s. The outside circle with different colors indicates different families of structural genes associated with Epimedin C or total PFGs biosynthesis in the blue module. The inside circles with different colors indicate different families of TFs characterized in the same module whose transcripts are highly correlated with the expression of structural genes.

WGCNA analysis was employed to construct the co-expression network to further test whether the predicted TFs were involved in PFGs accumulation. A total of 21,345 expressed genes (average TPM > 0.5) were clustered into 18 modules comprising 146~2,240 genes, and each module harbored TFs varying from 3 to 199 **(** **Figure 8A**, **Supplemental Table 13** **)**. Based on the correlation analysis between the module eigengene and the abundance of four PFGs (Epimedin A, Epimedin B, Epimedin C, and icariin) and total PFG content, the blue and brown module was significantly positively and negatively correlated with Epimedin C and total PFGs content, respectively **(** **Figure 8A** **)**. We selected blue module (containing 2,240 genes) for further analysis given that most genes relevant to PFGs biosynthesis came from this module.

In the blue module, we firstly extracted a subnetwork comprising of 1,898 genes, and identified 126 TFs and 37 PFGs biosynthesis genes, and then we filtered by TOM value or weight (threshold: 0.24), at which value, the maximum PFGs biosynthesis genes can be retained. The subnetwork (containing 650 genes) was further filtered by threshold of |MM| > 0.8 and |GS| > 0.2 (MM: module membership or eigengene-based connectivity, GS: gene significance). Finally, we completed network construction related to Epimedin C or total PFG accumulation and hub gene identification **(** **Figure 8B** **)**.

The core co-expression network contained 52 TFs, which belonged to 18 families, typified by MYB (12 genes), bHLH (6 genes), WD40 (8 genes), and bZIP (5 genes). Hub genes were those with higher intramodular connectivity, which have been visualized as larger circles **(** **Figure 8B** **)**, including 5 MYBs, 1 bHLH, 1 WD40, 2 bZIPs, 1 BES1, 1 C2H2, 1 Trihelix, 1 HD-ZIP, and 1 GATA. Further examinations revealed almost all of the TFs existed in the 7 TO-GCNs of PFGs biosynthesis genes **(** **Figures 4** **–** **7**, **Supplemental Figures 8**–**10** **)**, which confirmed that these TFs affect PFG content accumulation by participating in the regulation of target PFGs biosynthesis genes. In addition, almost all of the nine causal PFGs biosynthesis genes were included in this core network **(** **Figure 8B**, **Supplemental Table 8** **)**. This confirmed the reliability of the established TO-CNs network.

### Verification of gene regulatory relationships by qRT-PCR

**Figure 9:**
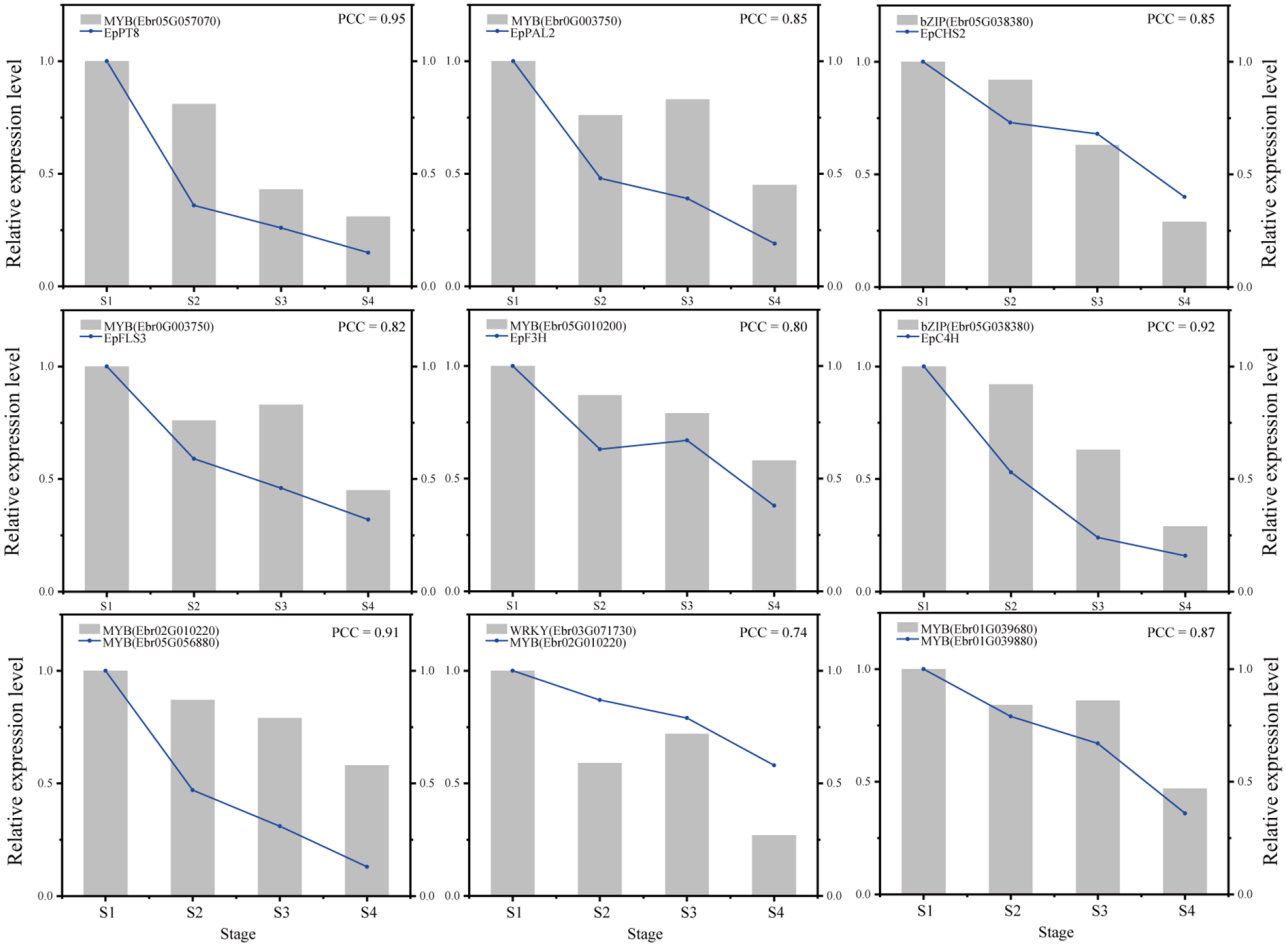
Verification of genes expression and regulatory relationships by qRT-PCR.

Nine gene pairs including TFs and their predicted target genes were verified by qRT-PCR (see the primers in **Supplemental Table 14** **)**. These gene pairs included *MYB* (*Ebr05G057070*) and *EpPT8*; *MYB* (*Ebr0G003750*) and *EpPAL2*; *bZIP* (*Ebr05G038380*) and *EpCHS2*; *MYB* (*Ebr0G003750*) and *EpFLS3*; *MYB* (*Ebr02G010220*) and *EpF3H*; *bZIP* (*Ebr05G038380*) and *EpC4H*; *MYB* (*Ebr02G010220*) and *MYB* (*Ebr05G056880*); *WRKY* (*Ebr03G071730*) and *MYB* (*Ebr02G010220*); and *MYB* (*Ebr01G039680*) and *MYB* (*Ebr01G039880*). The results showed that the gene pairs investigated exhibited strong correlations (Pearson correlation coefficient > 0.7), which further verified the reliability of the regulatory network **(** **Figure 9** **)**.

## Discussion

*Epimedium* herb has been widely used as important medicine due to its rare content of PFGs, which are known for their outstanding role in inhibiting hepatocellular carcinoma initiation and malignant growth. However, the biosynthesis and regulation mechanism of PFGs have not been systematically summarized and discussed with regard to *Epimedium*. Through high-temporal-resolution transcriptome and metabolome (targeted to PFGs) analysis during early leaf development, the molecular mechanism of PFGs accumulation and its regulation can be unraveled.

### Metabolic profiling differences of PFGs between buds and leaves in *Epimedium*

*Epimedium* species are herbaceous perennials grown from woody rhizomes, in which the meristem of buds triggered the emergence of leaves and flowers. A few studies have conducted the metabolic profiling on leaves, stems, and rhizomes (Xu et al., 2007; Yu J et al., 2012; Zhou M et al., 2021). However, there are no reports on buds. In this study, a significantly different metabolic profiling of PFGs was detected between buds and leaves **(** **Table 1** **)**. The main PFG in buds was Epimedoside A (demethylanhydroicaritin backbone, C-4′ linked hydroxyl), which was apparently higher than that in leaves; this is similar to the stem and rhizome reported by Zhou M et al. (2021). Ikarisoside A and 2′-*O*-rhamnosyl-Ikarisoside A (backbone of Type II) were only detected in buds. The main PFGs in leaves were dominant by Epimedin C and icariin (anhydroicaritin backbone, C-4′ linked methoxy) **(** **Table 1**, **Supplemental Table 1** **)**. Different *O*-methylation modification constituted the chemical diversity between buds and leaves. The higher enzyme activity of 4′-OMT in leaves may be the possible molecular basis, which can provide an explanation of the spatial distributions of metabolites and their chemical structures. Similar studies, e.g. the differential distribution of compounds in aerial and underground *Scutellaria baicalensis* (Zhao et al., 2016) and biosynthesis-based spatial metabolome of *Salvia miltiorrhiza*, have been in-depth discussed in depth (Tong et al., 2022).

### Possible reasons for the decrease of PFGs contents

Flavonoids have a strong antioxidant capacity and play an important role in promoting growth and development, which function at cellular-level processes, including cell division, membrane integrity, and ROS scavenging (Yadav et al., 2018). The significant metabolic flow transition for different stages of leaf development (S1~S4) **(** **Supplemental Figure 5**, **Supplemental Table 6** **)** suggested that the declined tendency of PFGs may be closely related to the demand for antioxidant ability and plant protection in young leaves. Studies of flavonoids in leaves of *Amygdalus pedunculata* (He et al., 2021), *Cistus ladanifer* (Valares Masa et al., 2016), and *Ginkgo biloba* (Wang et al., 2022) provide further evidence. In addition to S1, many biological processes towards “cell wall biosynthesis” were enriched in S2~S4, therefore, we deduced that with leaves aged, the increase in cell wall flavonoids may be paralleled by a decrease in soluble flavonoids in *Epimedium*. Since flavonoids are synthesized *via* a multienzyme complex localized in the endoplasmic reticulum (Zhu et al., 2016), the flavonoid transport deliver system in S1 may experience an efficient transport to each membrane-limited compartments, including nucleus and chloroplast. S2~S4 may occur vacuolar efflux of these soluble flavonoids and deposited in the cell wall (Zhao and Dixon, 2010). Further experiment with confocal laser scanning microscopy can provide more direct evidence.

PFGs biosynthesis genes constitute the direct reason for PFG contents variation. Transcriptome analysis showed that these genes, including *EpPAL2*, *EpCHS2*, *EpCHI2*, *EpF3H*, *EpFLS3*, and *EpPT8*, had expressions which were down-regulated gradually with leaf maturity **(** **Figure 3** **)**, which are in agreement with the metabolomics data, showing the highest PFG levels in S1 *Epimedium* leaves and its decrease during further leaf development. Similar results were observed in leaves of *E. sagitattum* (Huang et al., 2015), *A. pedunculata* (He et al., 2021), *C. ladanifer* (Valares Masa et al., 2016), and *G. biloba* (Wang et al., 2022).

In view of the collaborative expression of the PFGs biosynthetic genes, it could be presumed that these genes have been strictly regulated by TFs under temporal cues. This was in line with previous studies (Stracke et al., 2010; Naik et al., 2022). To explain the regulatory background of *Epimedium* PFGs metabolism during leaf development, we obtained 14 candidate TFs through WGCNA analysis **(** **Figure 8** **)**. It has been well established that SG7 MYB TFs contain SG7 motif ([K/R][R/x][R/K]xGRT[S/x][R/G]xx[M/x]K) and SG7-2 motif ([W/x][L/x]LS) at their C-termini, such as *AtMYB11*, *AtMYB12*, and *AtMYB111*, which function was direct activators to affect flavonol biosynthesis (Mehrtens et al., 2005; Stracke et al., 2007). The homologous genes have been investigated in *E. sagittatum* (Huang et al., 2015), grape (Czemmel et al., 2009), *Fagopyrum tataricum* (Yao et al., 2020), *Nicotiana tabacum* (Song et al., 2020), and pear (Zhai et al., 2019). Within the 14 TFs, one MYB *Ebr0G003750*, which was identified as one of the hub genes in co-expression network of Epimedin C or total PFGs, was the homolog of *EsMYBF1* identified in *E. sagittatum* (with 91.86% identity), which may be one of the direct activators involving in the regulating of PFG content. The consistency of the expression pattern of MYB *Ebr0G003750* with metabolomic data further verified this point. Other candidate MYB hub genes like MYB *Ebr05G057070* and *Ebr05G056880* (belonging to SG1, involved in environmental stress), and MYB *Ebr03G057220* (belonging to SG15, involving in epidermal cells) may involve indirect ways. MYB *Ebr03G023840* belongs to SG6 (involving in anthocyanin biosynthesis), and this gene may regulate both anthocyanin and flavonol biosynthesis pathways simultaneously. This type of gene has been reported in *Gerbera hybrida*, which is involved in the regulation of both flavonoids, as they share the same subcellular localization and common biosynthetic substrates, which may compete for substrates (Zhong et al., 2020).

### TO-GCN is conductive to discover new TF genes

We present here a TO-GCN approach to provide regulatory dynamics during leaf development. Compared to other time-series analysis method like Mfuzz (Kumar and Futschik, 2007), maSigPro (Conesa et al., 2006), and ImpulseDE2 (Fischer et al., 2018), TO-GCNs could predict upstream regulators of any genes in the GCNs (Chang et al., 2019). Given the important role of TFs as major drivers of genetic variation (Wallace et al., 2014), to understand which TFs control which sets of PFG biosynthesis genes, it is important for the rational metabolic engineering of plants with altered metabolites.

In general, it is much more difficult to predict an upstream regulator than a downstream target one. In this study, we revealed the cascade regulations of seven PFGs biosynthesis genes. Similar examples related to cascade regulation have been reported (Zhou et al., 2015; Li et al., 2020; Zhao et al., 2021). More than 1,022 TFs are assigned to the leaf development TO-GCN, providing a global picture of all gene regulatory relationships, and ~50 TFs were specially extracted through all 7 TO-GCNs related to PFG biosynthesis pathways (**Figure 4**–**7**, **Supplemental Figures 8**-**10**). This enriched our genetic resources with PFG regulation. In contrast, only one TF (*EsMYBF1*) (Huang et al., 2016a), two TFs (*EsAN2* and *EsMYBA1*) (Huang et al., 2012; Huang et al., 2013), and two TFs (*EsMYB7* and *EsMYB10*) (Huang et al., 2012) were identified as being involved in regulating flavonol, anthocyanin, and PAs biosynthesis in previous studies of *Epimedium*.

The TFs involved in flavonoid regulation have been reviewed (Li, 2014; Xu et al., 2015; Liu W et al., 2021). Besides the deeply studied *MYB11*, *MYB12*, and *MYB111*, which are functionally redundant and control the flavonol biosynthesis *via* activating the early biosynthetic genes such as *CHS*, *CHI*, *F3H*, *F3’H*, and *FLS*, many new TFs have been revealed, including activators *TCP3* (*bHLH*) (Li and Zachgo, 2013), *CsbZIP1* (Zhao et al., 2021), *VvibZIPc22* (Malacarne et al., 2016), and *AtWRKY23* (Grunewald et al., 2012), and repressors *CsPIF3* (Zhao et al., 2021), *FaMYB1* (Aharoni et al., 2001), and *BES1* (Liang et al., 2020). Based on TO-GCNs, we identified some unconfirmed TFs, which may be potential regulatory genes for flavonol synthesis. Li et al. (2021) reported that C2H2 and Trihelix indirectly promoted the synthesis of flavonoids by regulating abscisic acid (ABA) levels. The homolog genes of C2H2-type zinc finger (*Ebr0G014410*) and Trihelix DNA-binding factors (*Ebr01G020500*) were included in the TO-GCN of *EpFLS3*, and both genes positively correlated with the expression of *EpFLS3*, which may promote flavonol synthesis by reducing the inhibition of ABA on *EpFLS3* expression. Similarly, *BES1* (Liang et al., 2020) and *CsbZIP1* (Zhao et al., 2021) were recently reported, and these genes were included in the established TO-GCNs. It is reported that *BES1* served as a positive regulator in brassinosteroid signaling, inhibiting the transcription of *MYB11*, *MYB12*, and *MYB111*, thereby decreasing flavonol biosynthesis (Liang et al., 2020). In the tea plant, UV-B irradiation-mediated *bZIP1* upregulation leads to the promotion of flavonol biosynthesis by binding to the promoters of *MYB12*, *FLS*, and *UGT* and activating their expression (Zhao et al., 2021).

### Complex regulation mechanisms of PFGs biosynthesis were revealed in *Epimedium*

In this study, the interactivity of TFs with structural genes were highly complex, especially for genes like *EpPAL2* (49 TFs were predicted) and *EpCHS2* (43 TFs were predicted) **(** **Figures 5**, **6** **)**, which served as a control point of metabolic flow. Their *cis*-elements, including Silencer, H-box, ACE elements, AT-rich element, Box I and Box II, were regulated by many TFs and environmental factors (Liu et al., 2011; Pant and Huang, 2022). Genes like *EpPAL2*, *EpFLS3*, *EpC4H*, *EpCHS2*, *EpCHI2*, *EpF3H*, and *EpPT8* interacted with 11 or more regulatory partners, and expression of most of these genes was activated at S1~S2 or even before that **(** **Figures 4** **–** **7**, **Supplemental Figures 7**–**9** and **Supplemental Table 11** **)**. Our data suggested that gene interactions were at their highest complexity at the initiation of leaf, as has been described in petal color regulation in *R. simsii* (Yang F et al., 2020) and *S. oblata* (Ma et al., 2022).

We found almost all the PFG biosynthesis genes are co-regulated by a set of commonly shared TFs; this was evidenced in *CsMYBF1* (Liu et al., 2016), *GtMYBP3*, and *GtMYBP4* (Nakatsuka et al., 2012). Grotewold (2008) reported that the control of secondary metabolism is often carried out by TFs that are specialized in controlling particular branches of a pathway, often by activating or repressing the expression of a few genes encoding metabolic enzymes. The shared TFs in 7 TO-GCNs related to PFG biosynthesis pathways might the key regulators, including *MYB* (*Ebr02G010200*), *bZIP* (*Ebr05G038380*), *C2H2* (*Ebr05G010200*), *MYB* (*Ebr05G057070*), *MYB* (*Ebr0G003750*), *MYB* (*Ebr02G055930*), *MYB* (*Ebr01G039880*), and *WRKY* (*Ebr03G071730*) (**Figures 4** **–** **7**, **Supplemental Figure 7**–**9**). SG7 group MYBs, *Ebr0G003750*, *Ebr02G055930*, and *Ebr02G010200* can serve as the marker genes, which allowed us to find possible candidate pathways controlling flavonol pathway in *Epimedium*.

**Figure 10:**
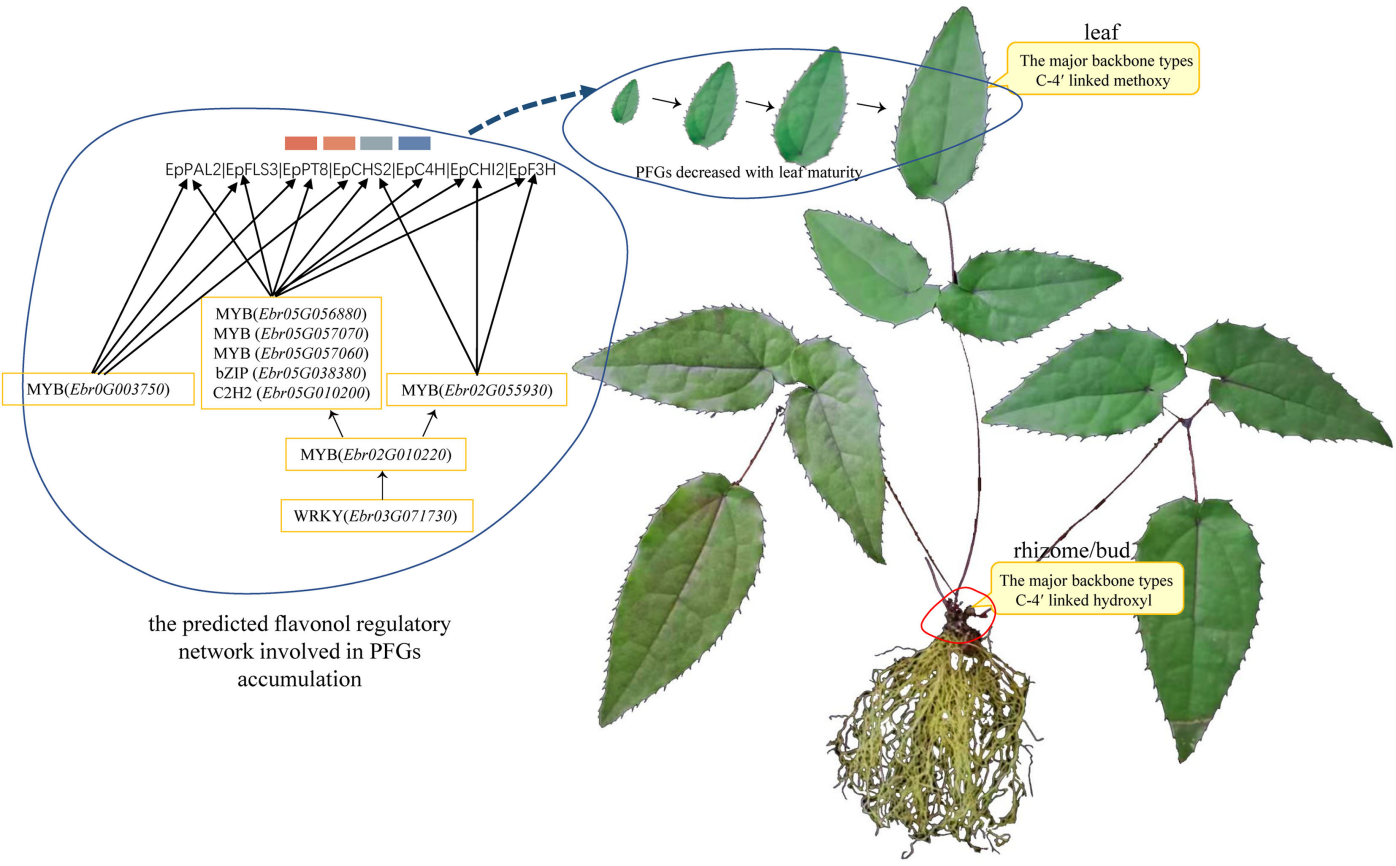
Possible model for this study.

Based on the aforementioned description, a model is proposed to elucidate the PFG accumulation pattern and how the aforementioned fine-tuners control flavonoid biosynthesis **(** **Figure 10** **)**. Although partial important TF and TF pairs or TF and biosynthesis genes relationships have been tested by qRT-PCR **(** **Figure 9** **),** the regulatory relationships between more TFs need to be further confirmed.

## Conclusion

By performing a combined analysis of metabolite profiling (targeted to prenylated flavonol glycosides) and a high-temporal-resolution transcriptome analysis in *E. pubescens*, the overall decline pattern of PFGs accumulation was clarified. Based on TO-GCNs, a TF gene homologous to *EsMYBF1* was found, multiple new TFs were predicted, and cascade regulatory networks related to prenylated flavonol glycosides were established. Partial TFs have been validated by WGCNA analysis and qRT-PCR. This is the first time that high-temporal-resolution transcriptome was performed to explore the cascade regulation of structural genes related to active ingredients accumulation in medicinal plants, which provide guidance for further studies on the role of TFs involved in PFGs biosynthesis and breeding programs. Although the TO-GCN results suggest the potential regulatory relationship between TFs and TFs, and between TFs and structural genes, further studies are needed in order to confirm the connections. In future work, molecular biology experiments such as subcellular localization and genetic transformation of hairy roots and yeast one-hybrid could be utilized to further verify the candidate TF activity in regulating the prenylated flavonol glycoside content and the binding ability to target gene promoters.

## Data availability statement

The datasets presented in this study can be found in online repositories. The names of the repository/repositories and accession number(s) can be found in the article/**Supplementary material**.

## Author contributions

BG and CX conceived and designed the study; CX, YZ, and XL prepared the materials, conducted the experiments, and analyzed all data; CX wrote the manuscript; XF, FS, GS, CS, BG, and CX were involved in data interpretation and finalizing the manuscript draft. All authors read and approved the final draft.

## Supplementary material

Supplementary Figure 1 Different UPLC-PDA fingerprint characteristics in different leaf development stages (S0~S5) of *E. pubescens.* (Rt=2.07 min) Diphyllodside B; (Rt=2.19 min) Epimedoside A; (Rt=4.11 min) Epimedin A; (Rt=4.32 min) Epimedin B; (Rt=4.52 min) Epimedin C; (Rt=4.71 min) icariin; (Rt=5.50 min) 3’’’-carbonyl-2’’-β-L-quinovosyl-icariin; (Rt=6.15 min) Anhydroicaritin-3-*O*-(acetyl) rhamnopyranosyl-xylopyranosyl-7-*O*-glucopyranoside; (Rt=7.36 min) 2’’-*O*-rhamnosyl-ikarisoside A; (Rt=7.42 min and Rt=7.46 min) Anhydroicaritin-3-*O*-(acetyl) rhamnopyranosyl-(acetyl)xylopyranosyl-7-*O*-glucopyranoside or its isomers; (Rt=7.53 min) Ikarisoside A; and (Rt=8.26 min) 2’’-*O*-rhamnopyranosyl icariside II; (Rt=8.77 min) icariside II.

Supplementary Figure 2 Mass spectrogram of chromatographic notable peak. Each PFG was identified under negative- and positive ion mode. **(A)** Diphyllodside B; **(B)** Epimedoside A; **(C)** Epimedin A; **(D)** Epimedin B; **(E)** Epimedin C; **(F)** icariin; **(G)** 3’’’-carbonyl-2’’-β-L-quinovosyl-icariin; **(H)** Anhydroicaritin-3-*O*-(acetyl)rhamnopyranosyl-xylopyranosyl-7-*O*-glucopyranoside; **(I)** 2’’-*O*-rhamnosyl-ikarisoside A; **(J)** Anhydroicaritin-3-*O*-(acetyl) rhamnopyranosyl-(acetyl)xylopyranosyl-7-*O*-glucopyranoside or its isomers; **(K)** Ikarisoside A; **(L)** 2’’-*O*-rhamnopyranosyl icariside II; **(M)** icariside II.

Supplementary Figure 3 Mother nucleus structure of PFGs in all stages of *E. pubescens* leaves.

Supplementary Figure 4 Comparative analysis of biological processes by GO enrichment in multiple leaf development stages.

Supplementary Figure 5 GO enrichment of S1 *vs* S4 up-regulated genes.

Supplementary Figure 6 KEGG enrichment of S1 *vs* S4 up-regulated genes.

Supplementary Figure 7 Classification of 4CL genes of *E. pubescens* determined by the classification system of *A. thaliana*.

Supplementary Figure 8 Resolved hierarchical regulation for *EpC4H*. The notion is the same as .

Supplementary Figure 9 Resolved hierarchical regulation for *EpCHI2*. The notion is the same as .

Supplementary Figure 10 Resolved hierarchical regulation for *EpF3H*. The notion is the same as.
