# Supplementary figures and images for: Time-series transcriptome provides insights into the gene regulation network involved in the icariin-flavonoid metabolism during the leaf development of Epimedium pubescens
Source: Front Plant Sci. 2023 Jun 12;14:1183481. doi: 10.3389/fpls.2023.1183481 (PMC10291196; doi:10.3389/fpls.2023.1183481)

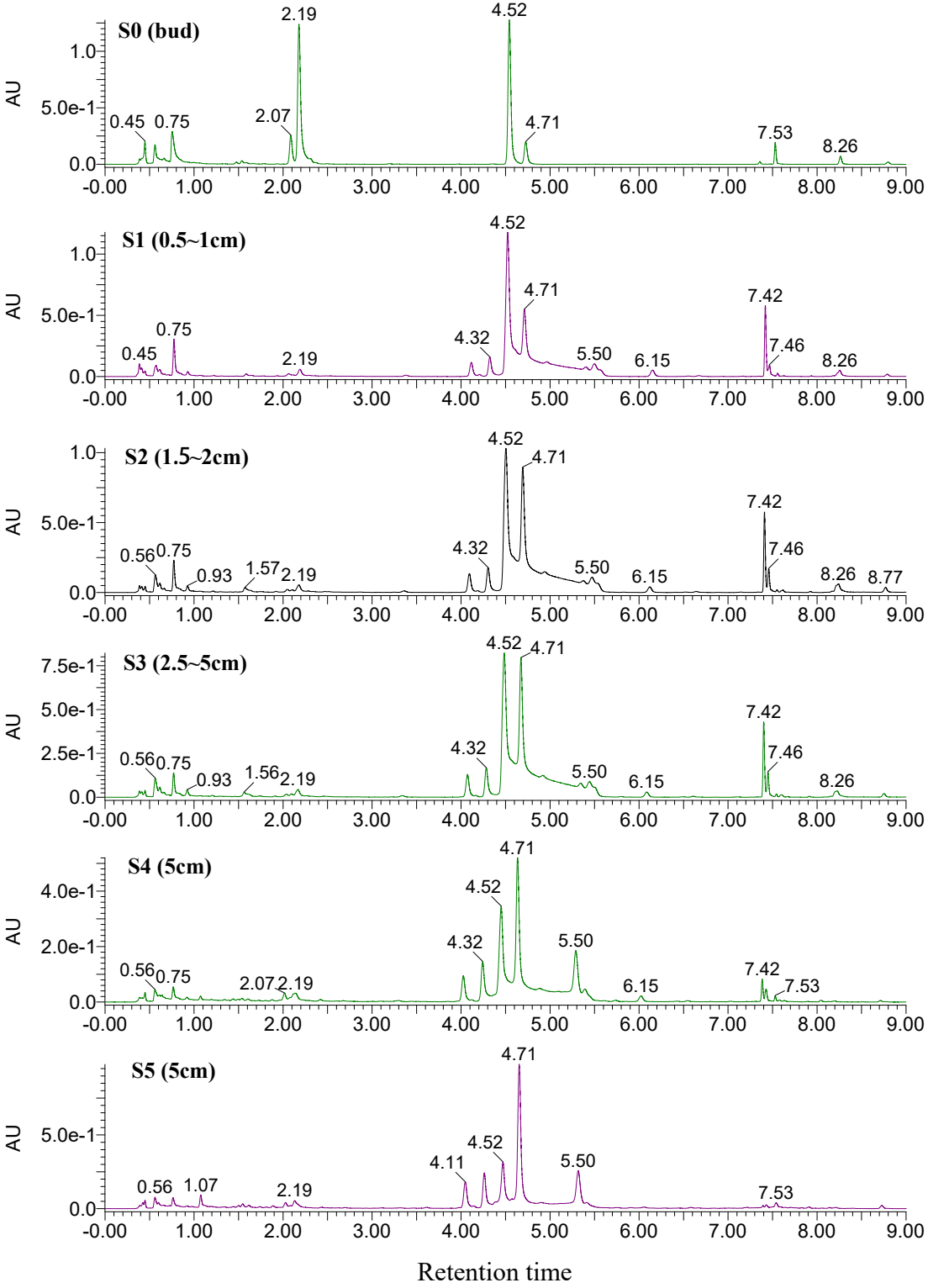

Supplement: Supplementary Figure 1 — Different UPLC-PDA fingerprint characteristics in different leaf development stages (S0~S5) of E. pubescens. (Rt=2.07 min) Diphyllodside B; (Rt=2.19 min) Epimedoside A; (Rt=4.11 min) Epimedin A; (Rt=4.32 min) Epimedin B; (Rt=4.52 min) Epimedin C; (Rt=4.71 min) icariin; (Rt=5.50 min) 3’’’-carbonyl-2’’-β-L-quinovosyl-icariin; (Rt=6.15 min) Anhydroicaritin-3-O-(acetyl) rhamnopyranosyl-xylopyranosyl-7-O-glucopyranoside; (Rt=7.36 min) 2’’-O-rhamnosyl-ikarisoside A; (Rt=7.42 min and Rt=7.46 min) Anhydroicaritin-3-O-(acetyl) rhamnopyranosyl-(acetyl)xylopyranosyl-7-O-glucopyranoside or its isomers; (Rt=7.53 min) Ikarisoside A; and (Rt=8.26 min) 2’’-O-rhamnopyranosyl icariside II; (Rt=8.77 min) icariside II. [file DataSheet_1.pdf]

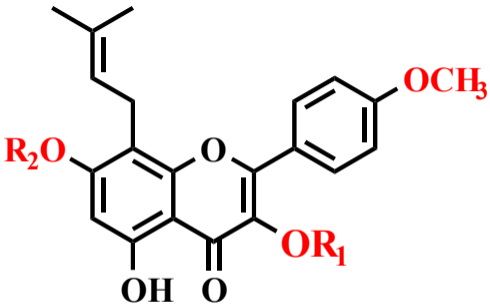

Type I

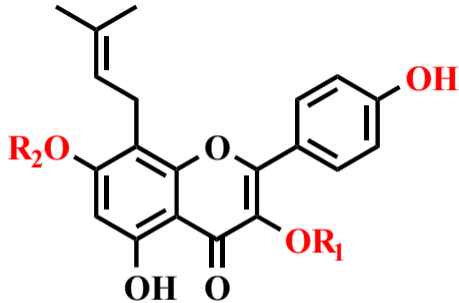

Type II

Supplement: Supplementary Figure 3 — Mother nucleus structure of PFGs in all stages of E. pubescens leaves. [file DataSheet_3.pdf]

# Enriched GO Terms (S1 vs S4 up-regulated)

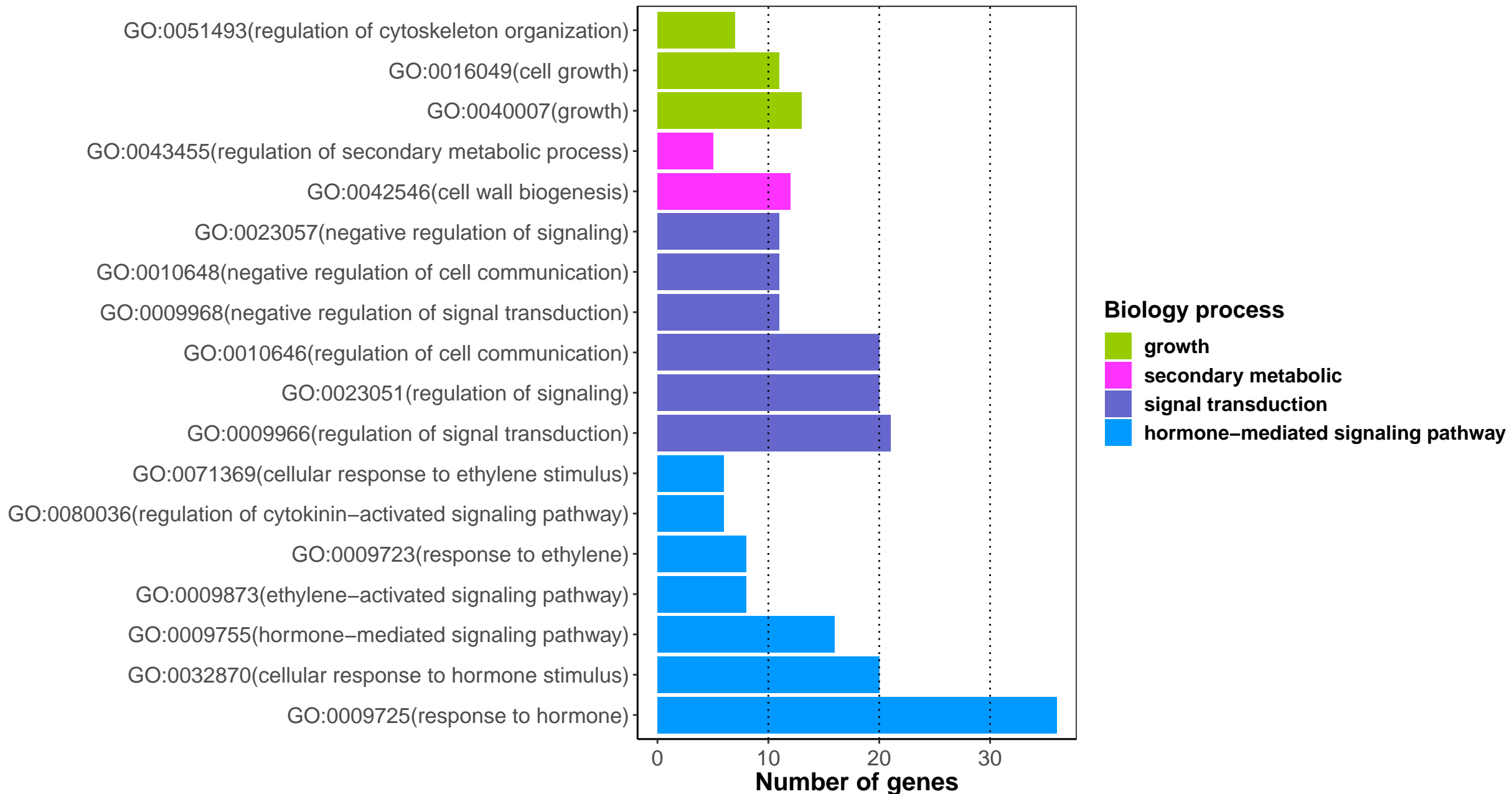

Supplement: Supplementary Figure 5 — GO enrichment of S1 vs S4 up-regulated genes. [file DataSheet_5.pdf]

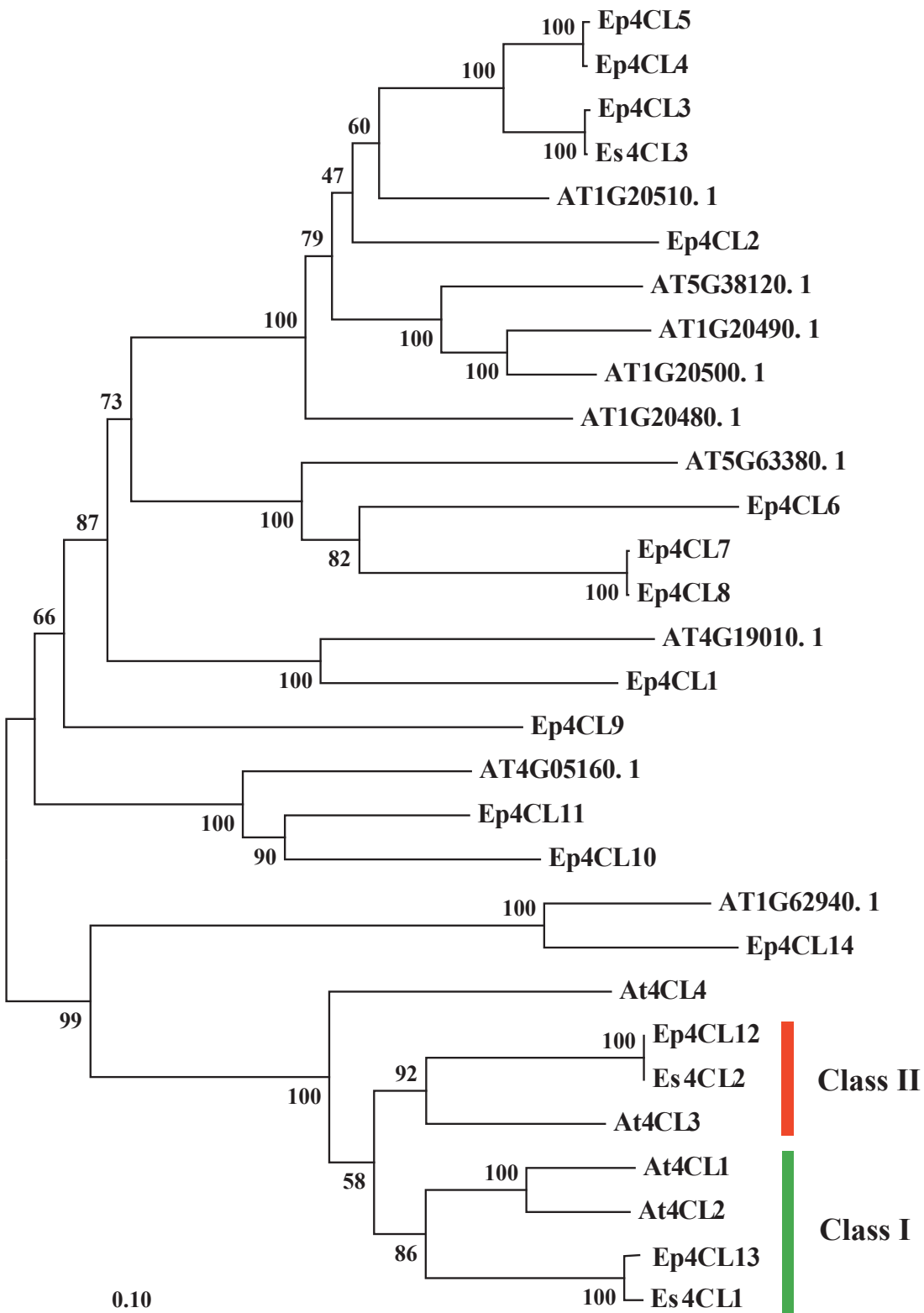

Supplement: Supplementary Figure 7 — Classification of 4CL genes of E. pubescens determined by the classification system of A. thaliana. [file DataSheet_7.pdf]

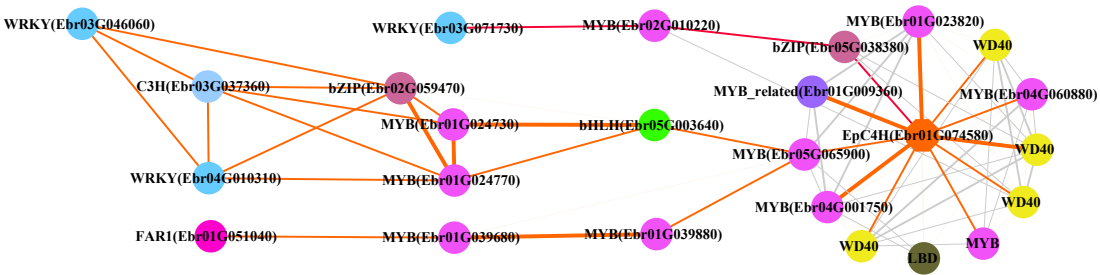

Supplement: Supplementary Figure 8 — Resolved hierarchical regulation for EpC4H. The notion is the same as . [file DataSheet_8.pdf]

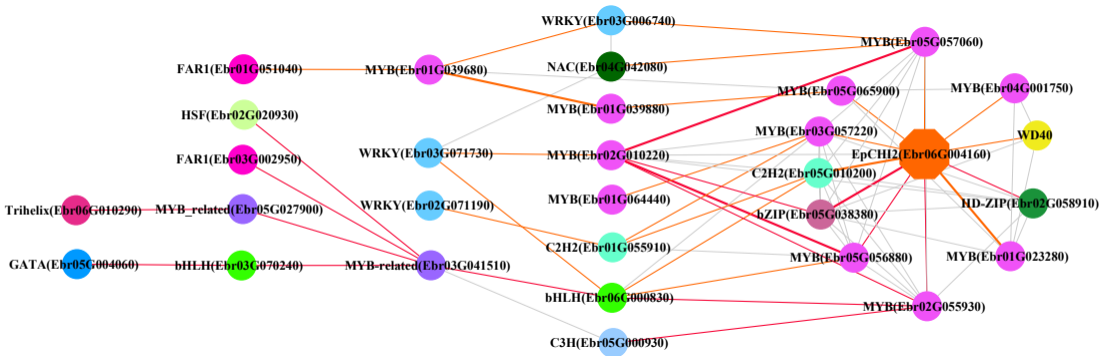

Supplement: Supplementary Figure 9 — Resolved hierarchical regulation for EpCHI2. The notion is the same as . [file DataSheet_9.pdf]

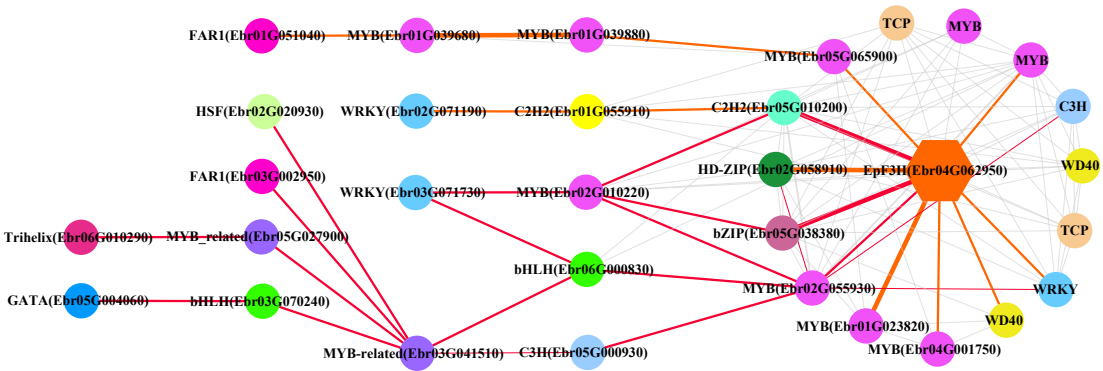

Supplement: Supplementary Figure 10 — Resolved hierarchical regulation for EpF3H. The notion is the same as. [file DataSheet_10.pdf]
