# Supplementary material for: Time-series transcriptome provides insights into the gene regulation network involved in the icariin-flavonoid metabolism during the leaf development of Epimedium pubescens
Source: Front Plant Sci. 2023 Jun 12;14:1183481. doi: 10.3389/fpls.2023.1183481 (PMC10291196; doi:10.3389/fpls.2023.1183481)
Supplement: Supplementary Figure 2 — Mass spectrogram of chromatographic notable peak. Each PFG was identified under negative- and positive ion mode. (A) Diphyllodside B; (B) Epimedoside A; (C) Epimedin A; (D) Epimedin B; (E) Epimedin C; (F) icariin; (G) 3’’’-carbonyl-2’’-β-L-quinovosyl-icariin; (H) Anhydroicaritin-3-O-(acetyl)rhamnopyranosyl-xylopyranosyl-7-O-glucopyranoside; (I) 2’’-O-rhamnosyl-ikarisoside A; (J) Anhydroicaritin-3-O-(acetyl) rhamnopyranosyl-(acetyl)xylopyranosyl-7-O-glucopyranoside or its isomers; (K) Ikarisoside A; (L) 2’’-O-rhamnopyranosyl icariside II; (M) icariside II. [file DataSheet_2.pdf]

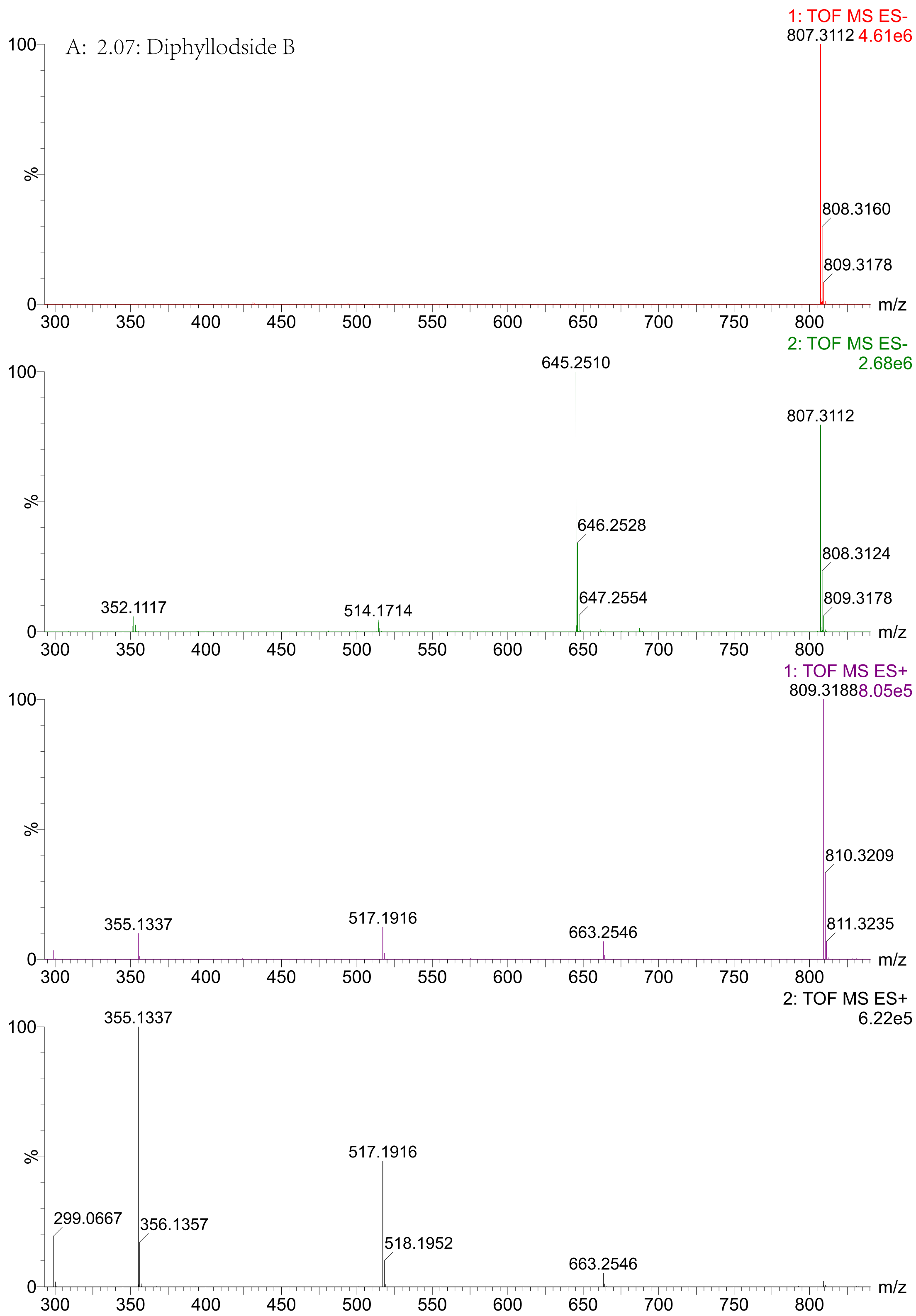
A: 2.07: Diphyllodside B
1: TOF MS ES-
807.3112 4.61e6
808.3160
809.3178
2: TOF MS ES-
2.68e6
645.2510
807.3112
646.2528
808.3124
647.2554
809.3178
352.1117
514.1714
1: TOF MS ES+
809.3188 8.05e5
810.3209
811.3235
355.1337
517.1916
663.2546
2: TOF MS ES+
6.22e5
355.1337
517.1916
299.0667
356.1357
518.1952
663.2546
%
m/z
100
0
300
350
400
450
500
550
600
650
700
750
800

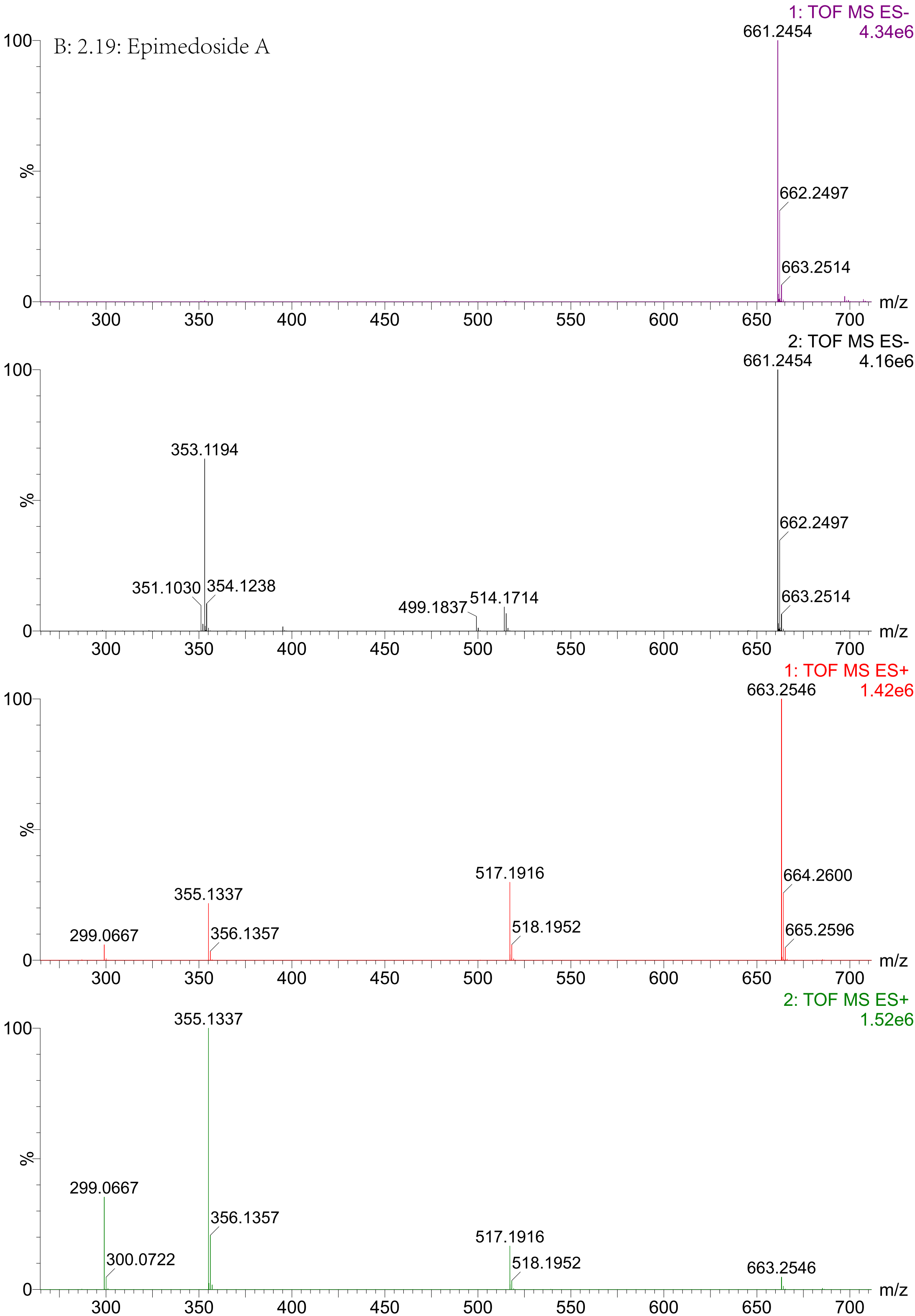
B: 2.19: Epimedoside A
1: TOF MS ES-
4.34e6
661.2454
662.2497
663.2514
2: TOF MS ES-
4.16e6
661.2454
353.1194
351.1030
354.1238
499.1837
514.1714
662.2497
663.2514
1: TOF MS ES+
1.42e6
663.2546
517.1916
355.1337
299.0667
356.1357
518.1952
664.2600
665.2596
2: TOF MS ES+
1.52e6
355.1337
299.0667
356.1357
300.0722
517.1916
518.1952
663.2546
%
m/z
100
0
300
350
400
450
500
550
600
650
700

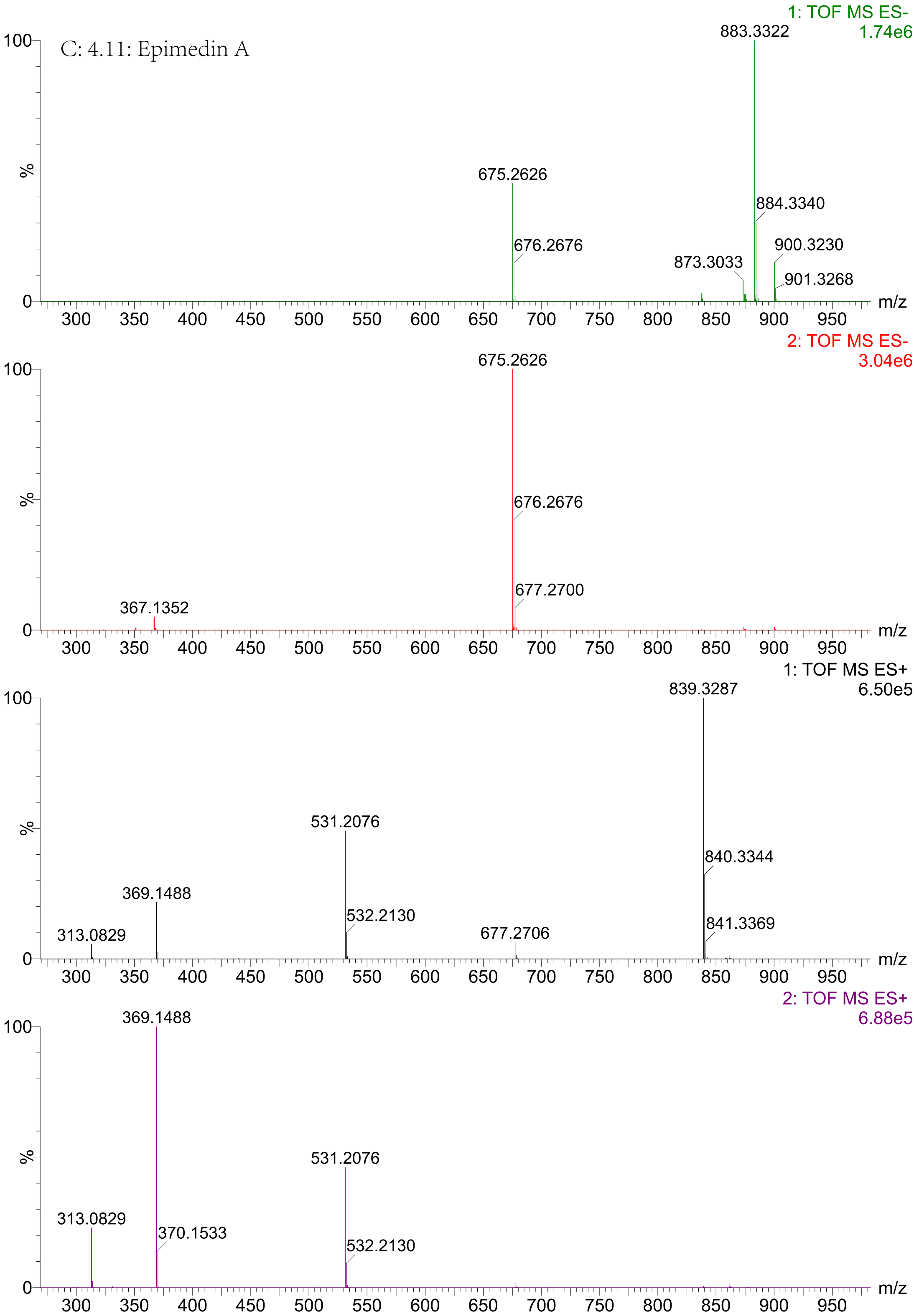

C: 4.11: Epimedin A
1: TOF MS ES-
1.74e6
883.3322
675.2626
676.2676
884.3340
900.3230
873.3033
901.3268
2: TOF MS ES-
3.04e6
675.2626
676.2676
677.2700
367.1352
1: TOF MS ES+
6.50e5
839.3287
531.2076
840.3344
369.1488
532.2130
841.3369
313.0829
677.2706
2: TOF MS ES+
6.88e5
369.1488
531.2076
313.0829
370.1533
532.2130
%
m/z

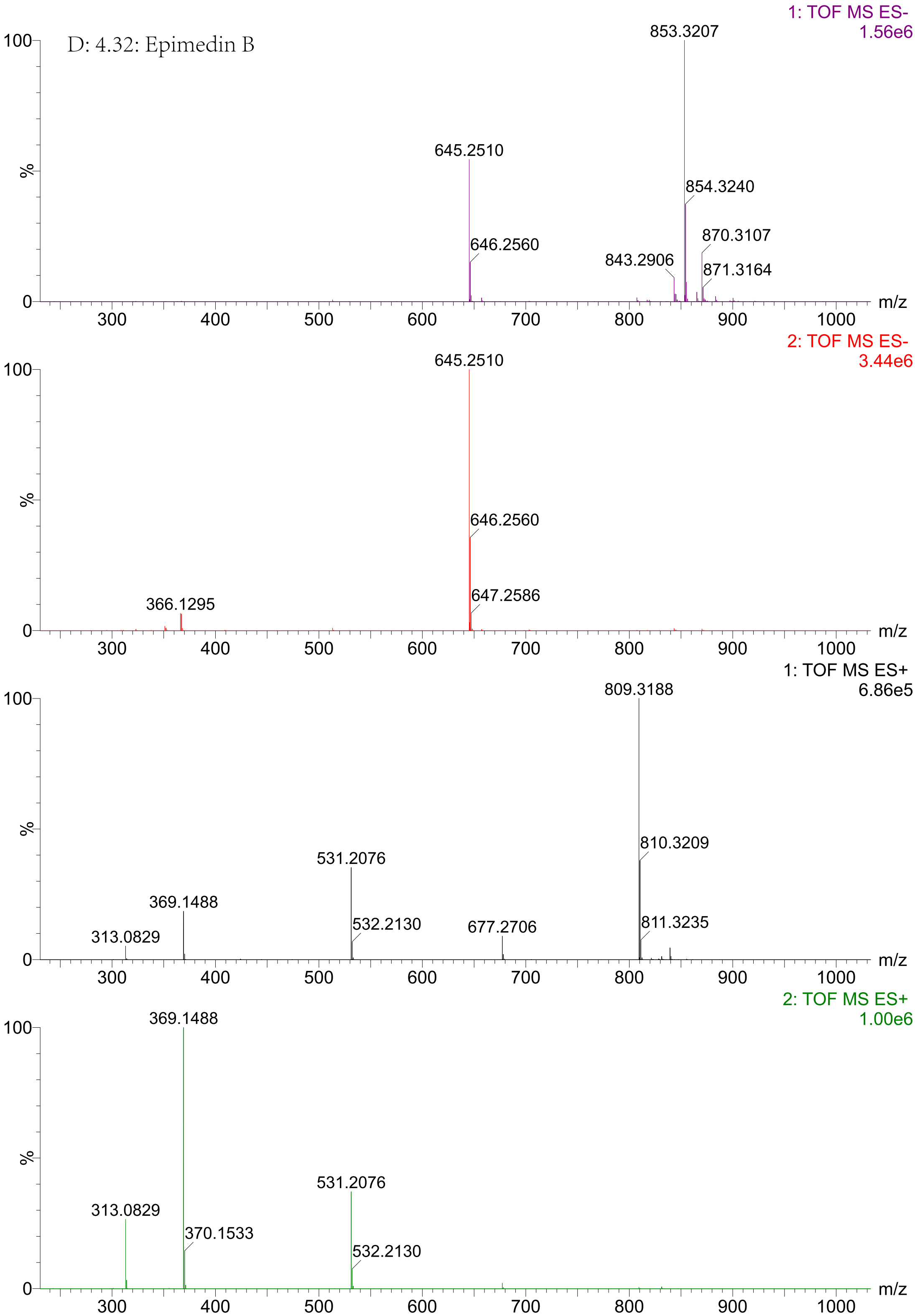
D: 4.32: Epimedin B
1: TOF MS ES-
1.56e6
853.3207
645.2510
854.3240
646.2560
870.3107
843.2906
871.3164
2: TOF MS ES-
3.44e6
645.2510
646.2560
647.2586
366.1295
1: TOF MS ES+
6.86e5
809.3188
810.3209
531.2076
369.1488
532.2130
677.2706
811.3235
313.0829
2: TOF MS ES+
1.00e6
369.1488
531.2076
313.0829
370.1533
532.2130
%
m/z
100
0
300
400
500
600
700
800
900
1000

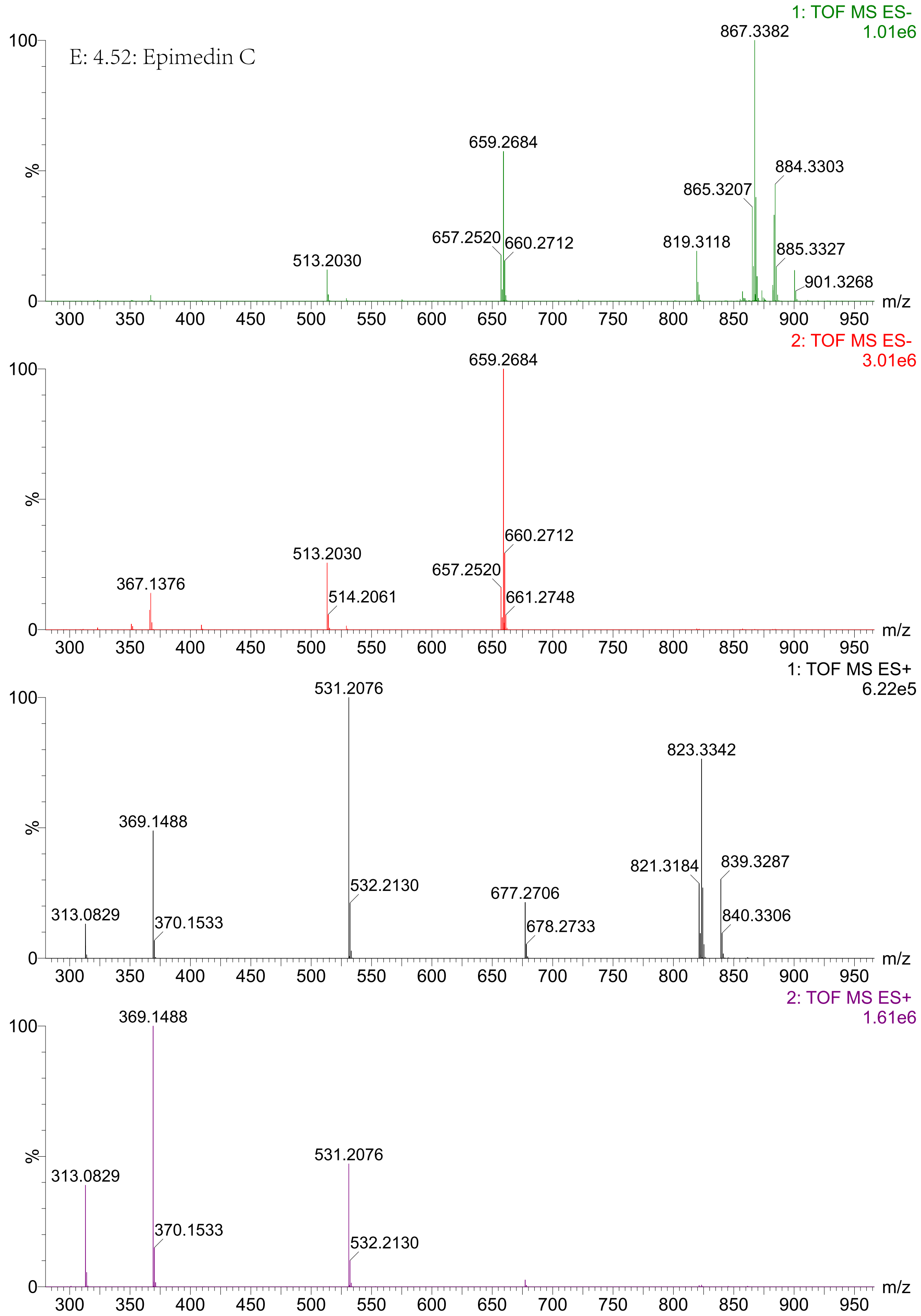

E: 4.52: Epimedin C
1: TOF MS ES-
1.01e6
867.3382
659.2684
884.3303
865.3207
657.2520
660.2712
819.3118
885.3327
513.2030
901.3268
2: TOF MS ES-
3.01e6
659.2684
660.2712
513.2030
657.2520
367.1376
514.2061
661.2748
1: TOF MS ES+
6.22e5
531.2076
823.3342
369.1488
821.3184
839.3287
532.2130
677.2706
313.0829
370.1533
678.2733
840.3306
2: TOF MS ES+
1.61e6
369.1488
531.2076
313.0829
370.1533
532.2130
100
%
0
m/z
300
350
400
450
500
550
600
650
700
750
800
850
900
950

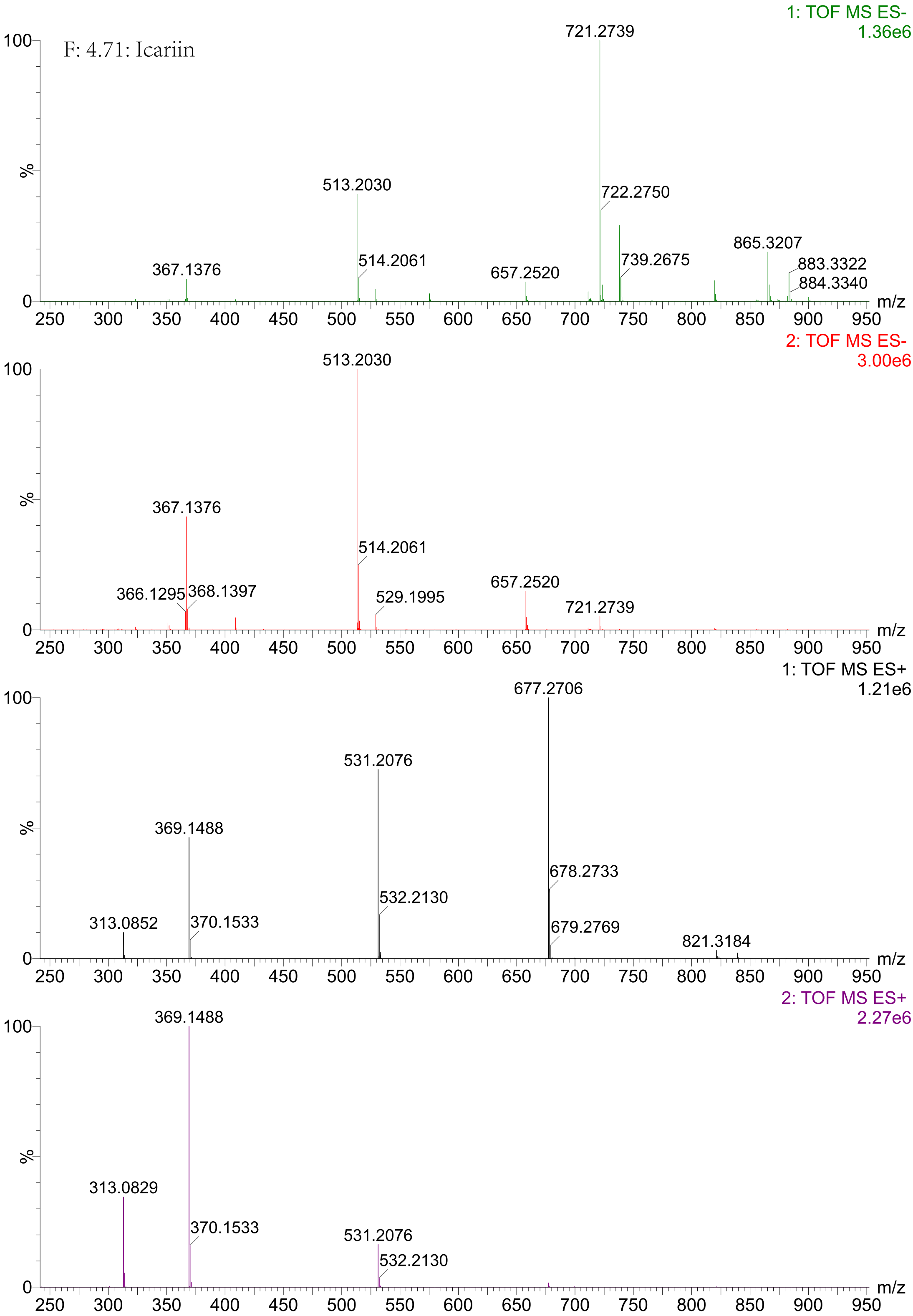

F: 4.71: Icariin
1: TOF MS ES-
1.36e6
721.2739
722.2750
739.2675
865.3207
883.3322
884.3340
657.2520
513.2030
514.2061
367.1376
2: TOF MS ES-
3.00e6
513.2030
367.1376
514.2061
366.1295
368.1397
529.1995
657.2520
721.2739
1: TOF MS ES+
1.21e6
677.2706
531.2076
369.1488
678.2733
532.2130
313.0852
370.1533
679.2769
821.3184
2: TOF MS ES+
2.27e6
369.1488
313.0829
370.1533
531.2076
532.2130
100
%
0
m/z
250
300
350
400
450
500
550
600
650
700
750
800
850
900
950

G: 5.50: 3'''-carbonyl-2''- β -L-quinovosyl-icariin

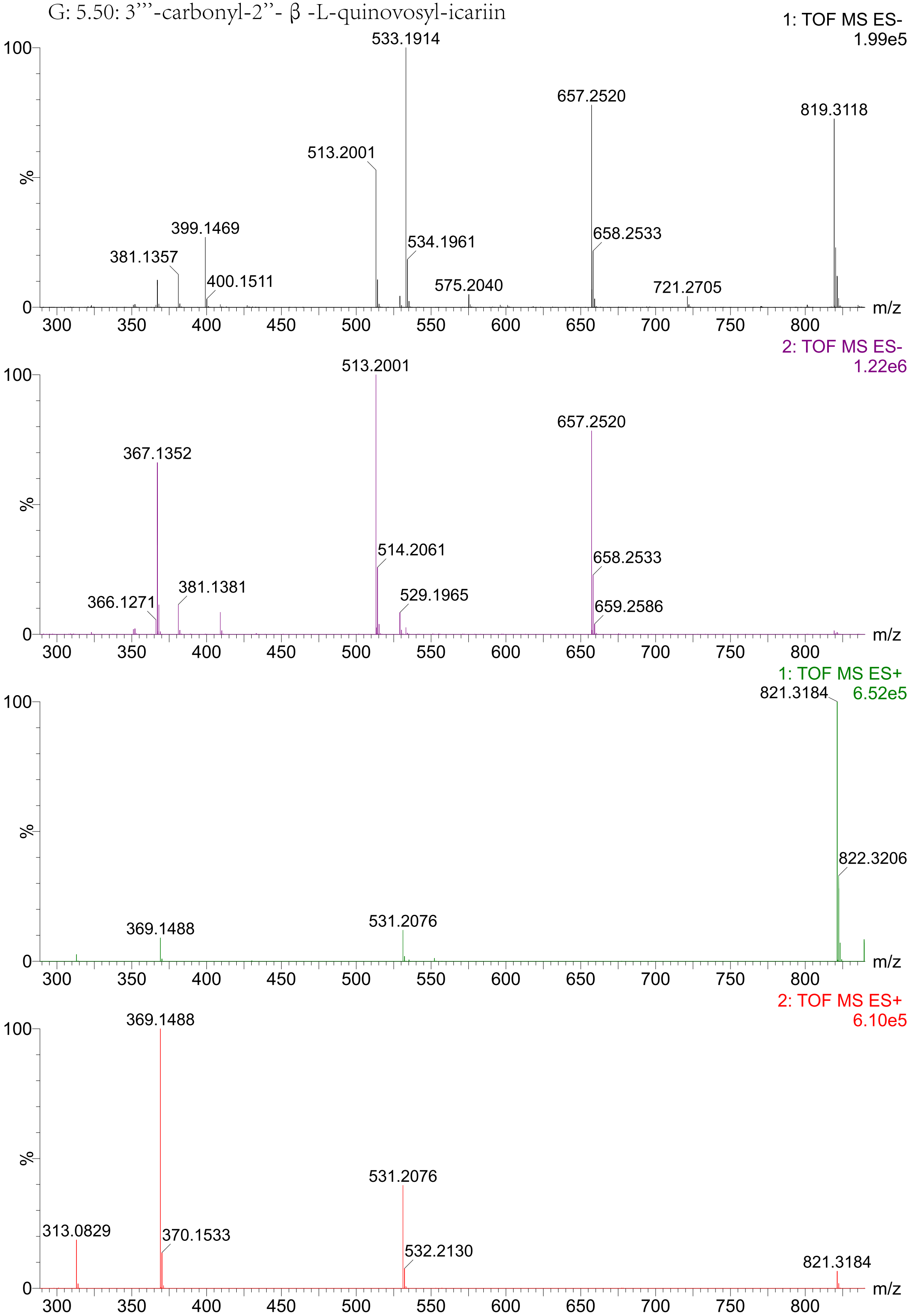

H: 6.15: Anhydroicaritin-3-O-(acetyl) rhamnopyranosyl-xylopyranosyl-7-O-glucopyranoside

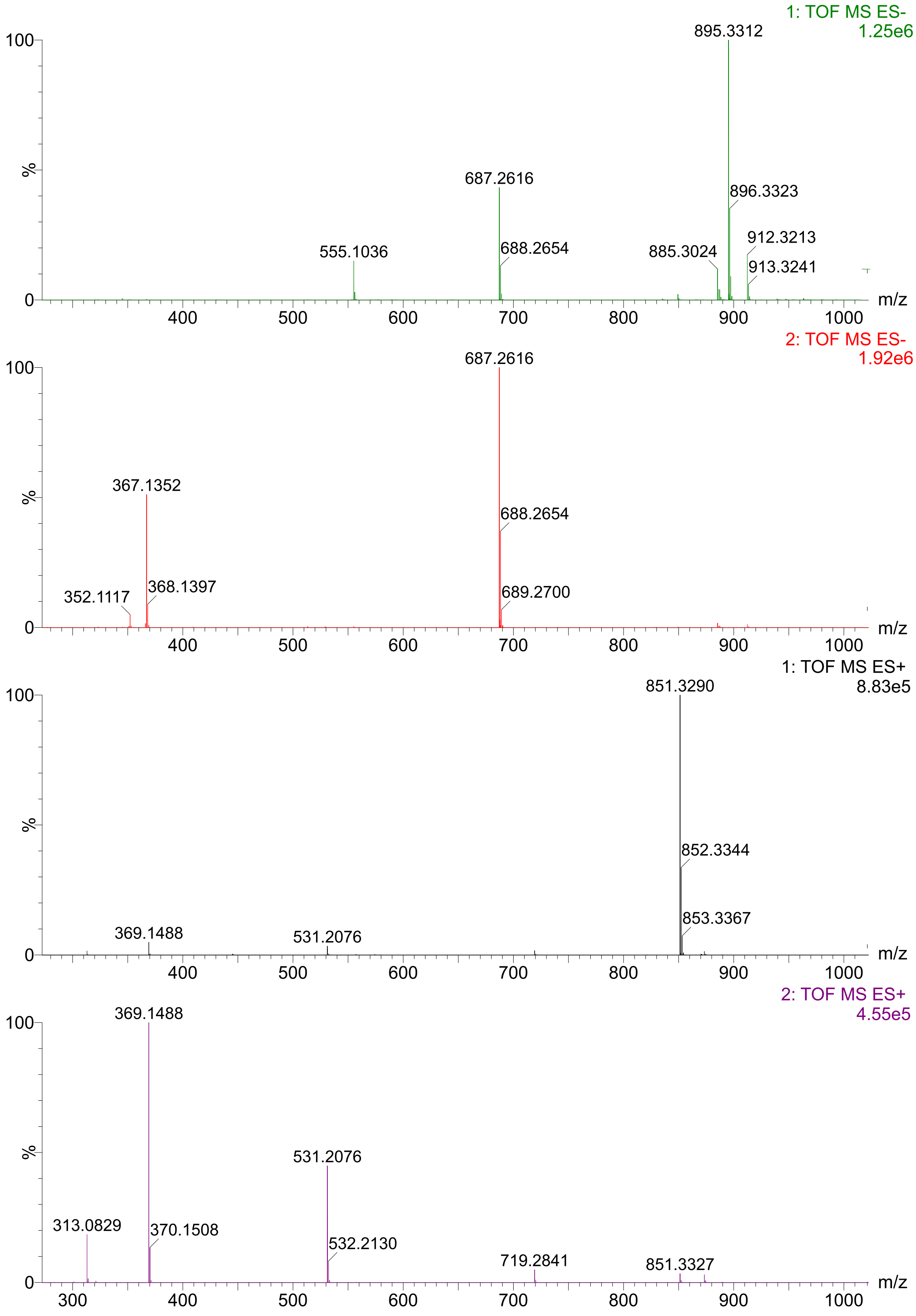

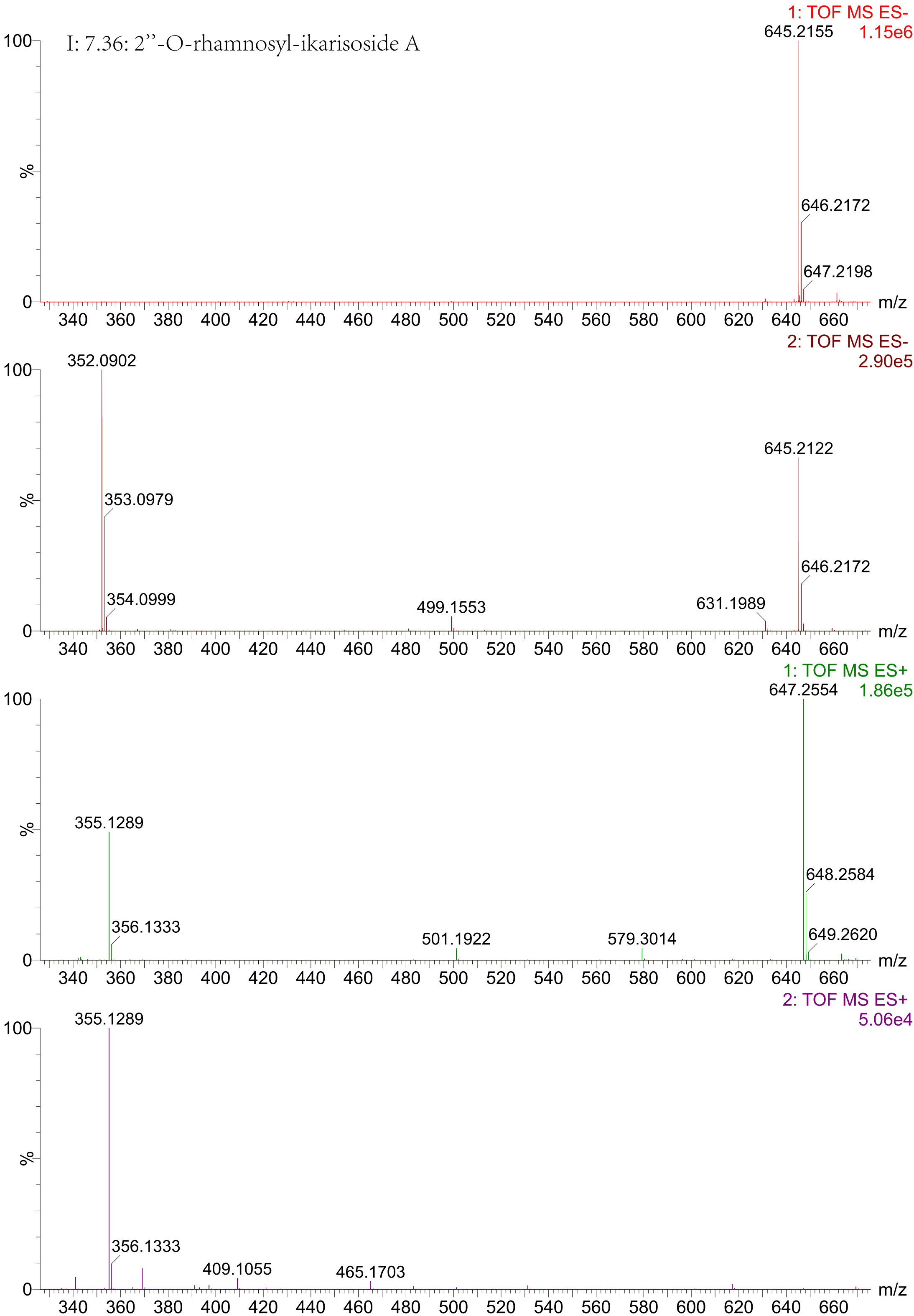

I: 7.36: 2''-O-rhamnosyl-ikarisoside A
1: TOF MS ES-
1.15e6
645.2155
646.2172
647.2198
2: TOF MS ES-
2.90e5
352.0902
353.0979
354.0999
499.1553
631.1989
645.2122
646.2172
1: TOF MS ES+
1.86e5
647.2554
355.1289
356.1333
501.1922
579.3014
648.2584
649.2620
2: TOF MS ES+
5.06e4
355.1289
356.1333
409.1055
465.1703
%
m/z

J: 7.42 and 7.46: Anhydroicaritin-3-O-(acetyl) rhamnopyranosyl-(acetyl)xylopyranosyl-7-O-glucopyranoside or its isomers

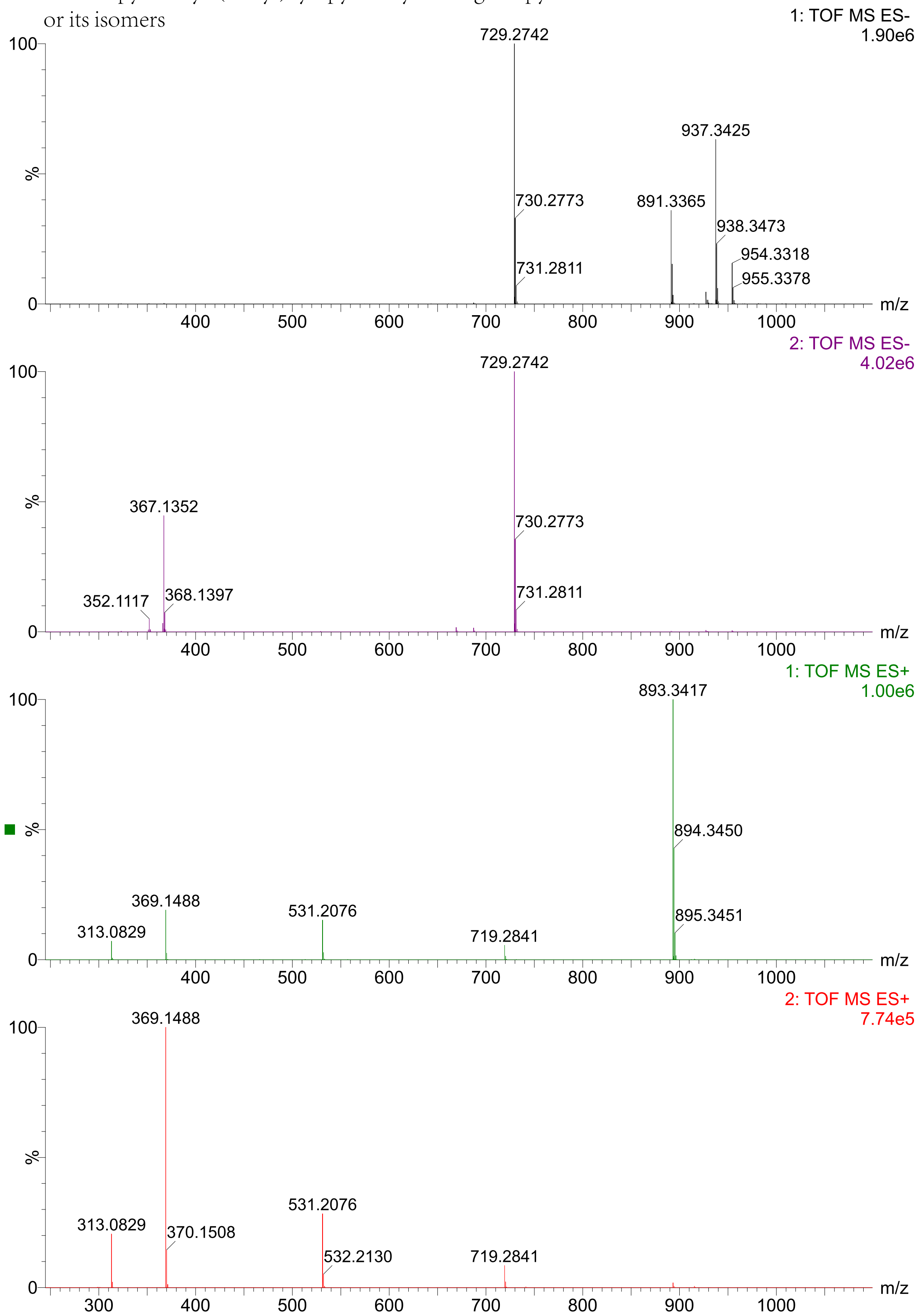

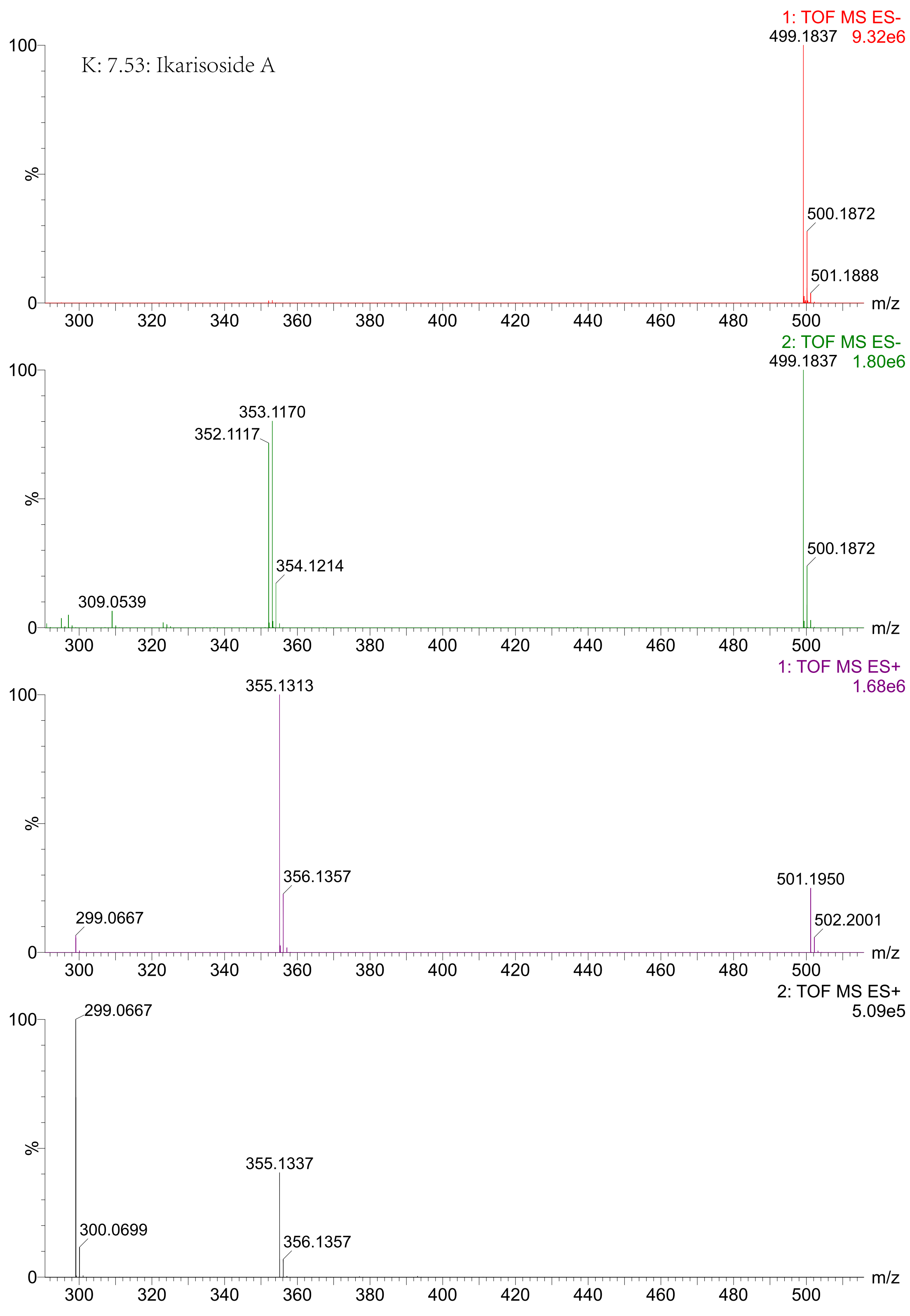
K: 7.53: Ikarisoside A
1: TOF MS ES-
9.32e6
499.1837
500.1872
501.1888
2: TOF MS ES-
1.80e6
499.1837
353.1170
352.1117
354.1214
309.0539
500.1872
1: TOF MS ES+
1.68e6
355.1313
356.1357
299.0667
501.1950
502.2001
2: TOF MS ES+
5.09e5
299.0667
300.0699
355.1337
356.1357
%
m/z

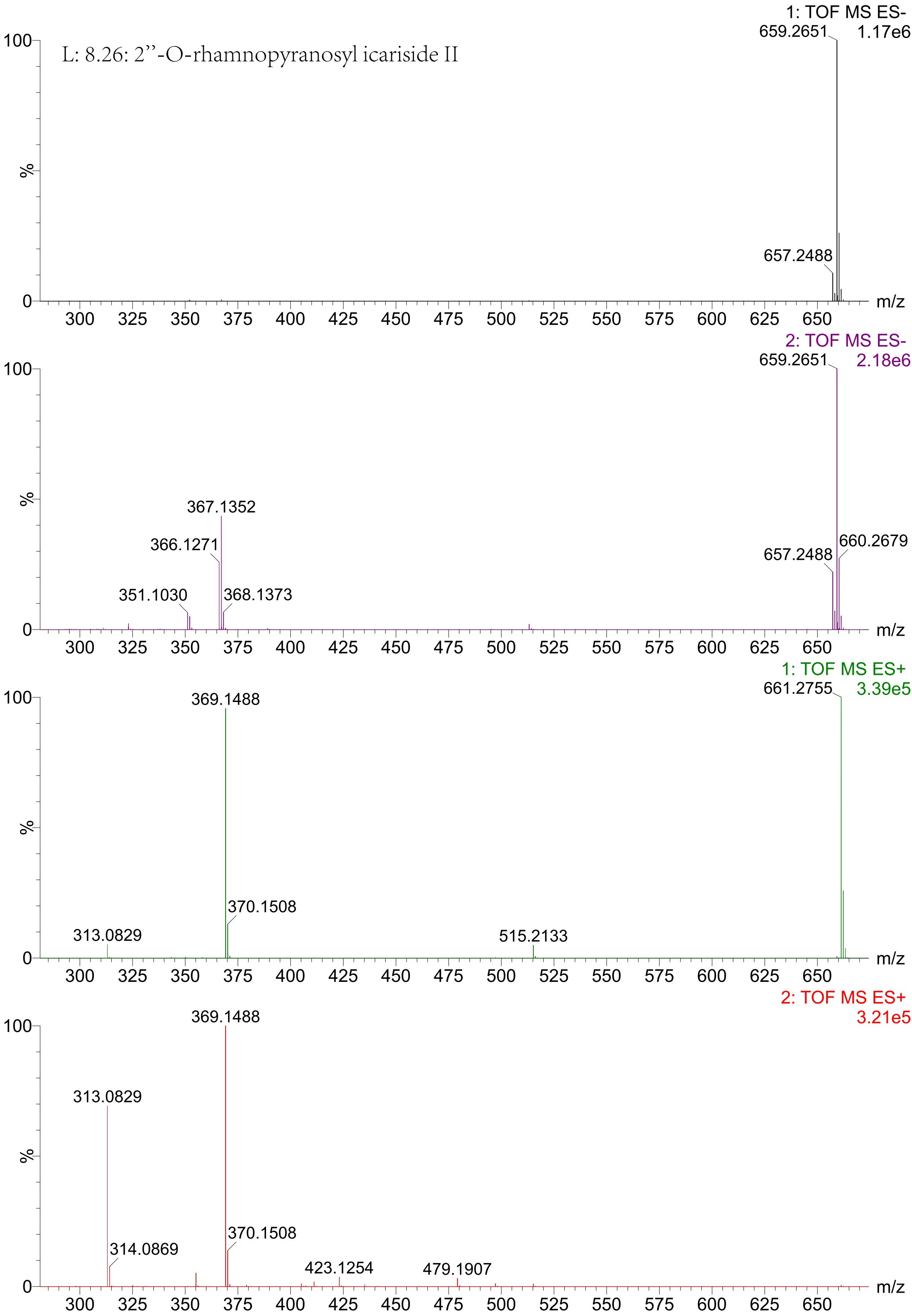

L: 8.26: 2”-O-rhamnopyranosyl icariside II
1: TOF MS ES-
1.17e6
659.2651
657.2488
100
%
0
m/z
300
325
350
375
400
425
450
475
500
525
550
575
600
625
650
2: TOF MS ES-
2.18e6
659.2651
367.1352
366.1271
660.2679
657.2488
351.1030
368.1373
1: TOF MS ES+
3.39e5
661.2755
369.1488
370.1508
313.0829
515.2133
2: TOF MS ES+
3.21e5
369.1488
313.0829
370.1508
314.0869
423.1254
479.1907

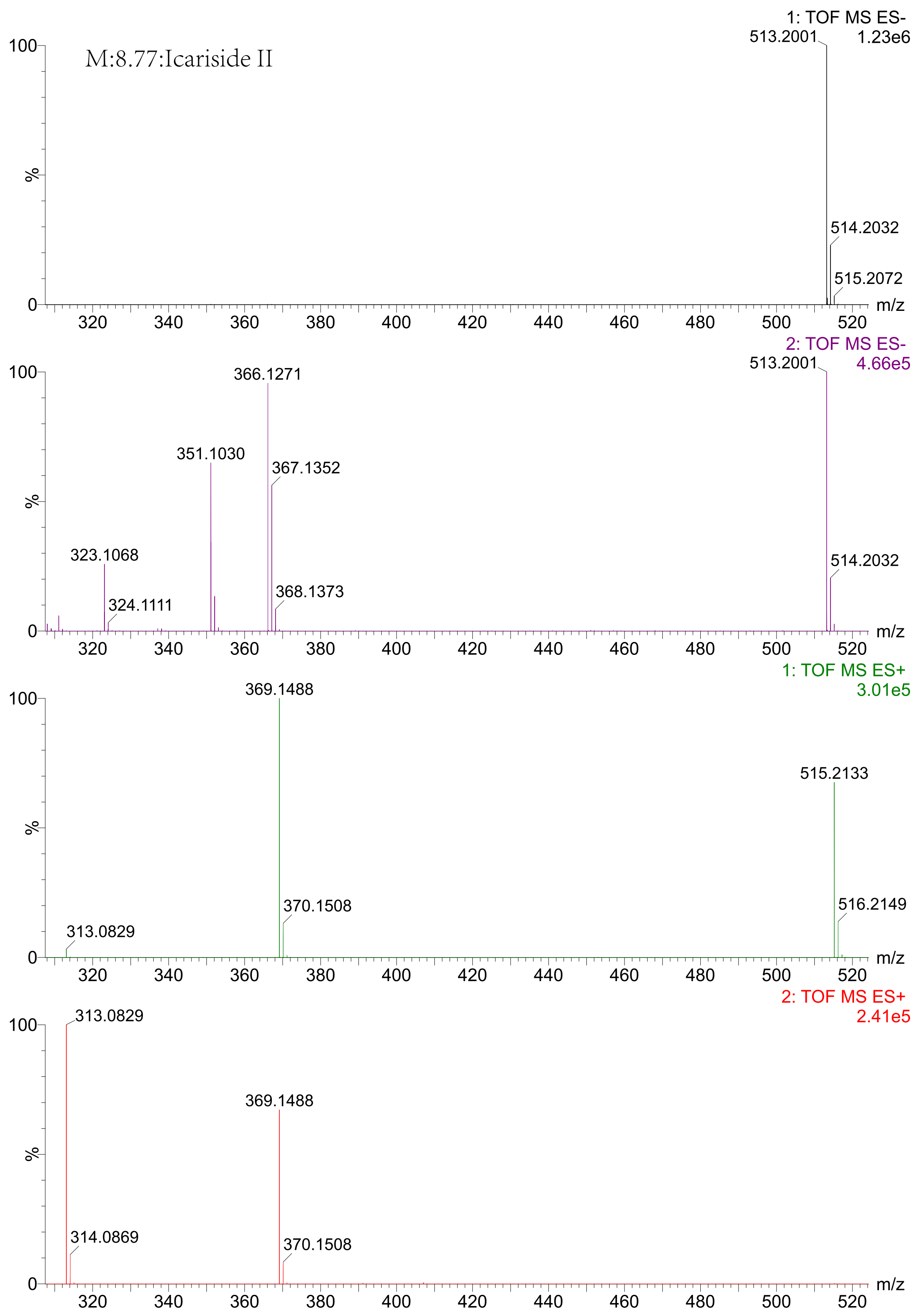

M:8.77:Icariside II
1: TOF MS ES-
1.23e6
513.2001
514.2032
515.2072
2: TOF MS ES-
4.66e5
513.2001
366.1271
351.1030
367.1352
323.1068
324.1111
368.1373
514.2032
1: TOF MS ES+
3.01e5
369.1488
515.2133
370.1508
516.2149
313.0829
2: TOF MS ES+
2.41e5
313.0829
369.1488
314.0869
370.1508
%
m/z
