# Supplementary material for: Time-series transcriptome provides insights into the gene regulation network involved in the icariin-flavonoid metabolism during the leaf development of Epimedium pubescens
Source: Front Plant Sci. 2023 Jun 12;14:1183481. doi: 10.3389/fpls.2023.1183481 (PMC10291196; doi:10.3389/fpls.2023.1183481)
Supplement: Supplementary Figure 4 — Comparative analysis of biological processes by GO enrichment in multiple leaf development stages. [file DataSheet_4.pdf]

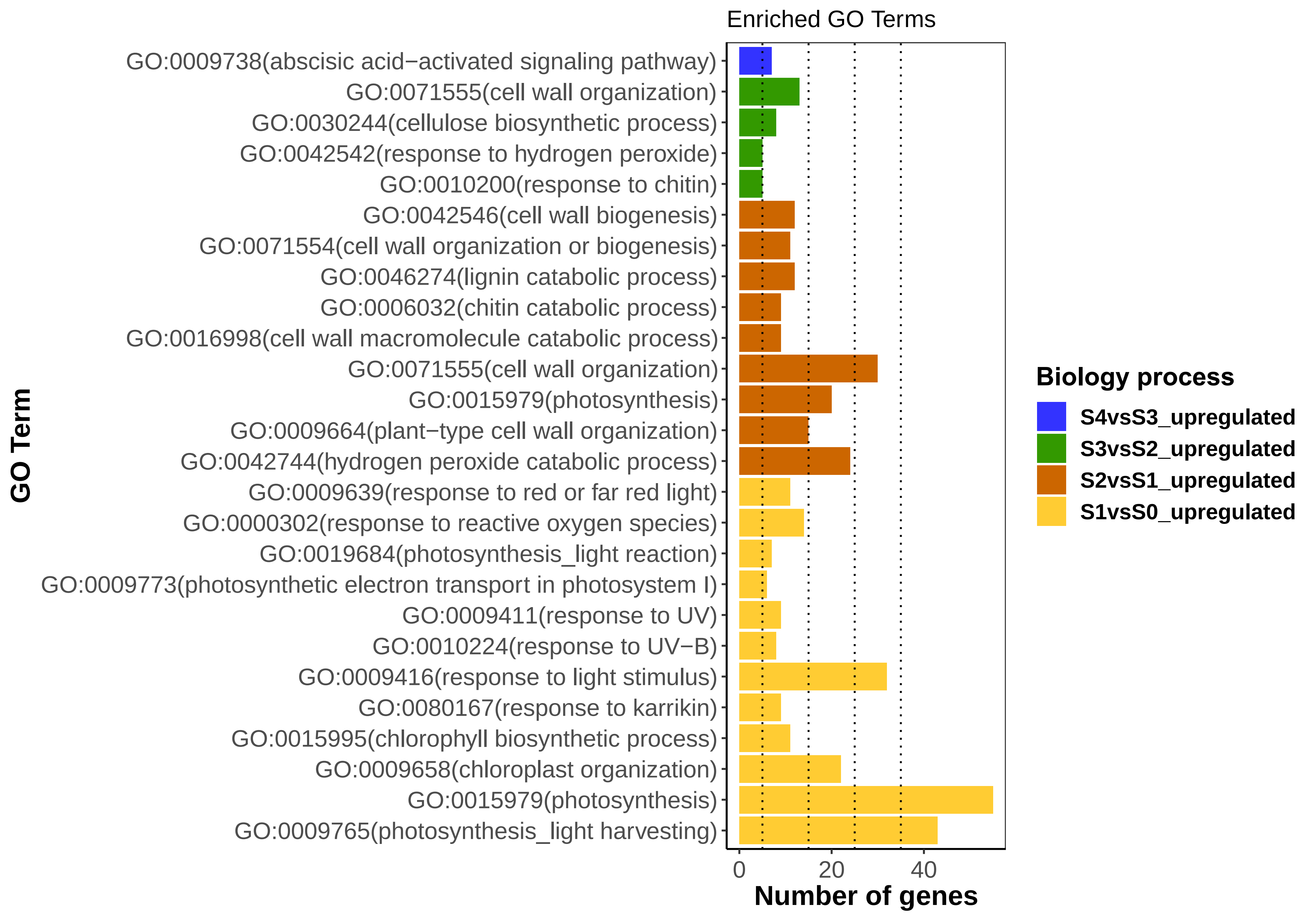

Enriched GO Terms
GO:0009738(abscisic acid−activated signaling pathway)
GO:0071555(cell wall organization)
GO:0030244(cellulose biosynthetic process)
GO:0042542(response to hydrogen peroxide)
GO:0010200(response to chitin)
GO:0042546(cell wall biogenesis)
GO:0071554(cell wall organization or biogenesis)
GO:0046274(lignin catabolic process)
GO:0006032(chitin catabolic process)
GO:0016998(cell wall macromolecule catabolic process)
GO:0071555(cell wall organization)
GO:0015979(photosynthesis)
GO:0009664(plant−type cell wall organization)
GO:0042744(hydrogen peroxide catabolic process)
GO:0009639(response to red or far red light)
GO:0000302(response to reactive oxygen species)
GO:0019684(photosynthesis_light reaction)
GO:0009773(photosynthetic electron transport in photosystem I)
GO:0009411(response to UV)
GO:0010224(response to UV−B)
GO:0009416(response to light stimulus)
GO:0080167(response to karrikin)
GO:0015995(chlorophyll biosynthetic process)
GO:0009658(chloroplast organization)
GO:0015979(photosynthesis)
GO:0009765(photosynthesis_light harvesting)
GO Term
0
20
40
Number of genes
Biology process
S4vsS3_upregulated
S3vsS2_upregulated
S2vsS1_upregulated
S1vsS0_upregulated
