# Supplementary material for: Time-series transcriptome provides insights into the gene regulation network involved in the icariin-flavonoid metabolism during the leaf development of Epimedium pubescens
Source: Front Plant Sci. 2023 Jun 12;14:1183481. doi: 10.3389/fpls.2023.1183481 (PMC10291196; doi:10.3389/fpls.2023.1183481)
Supplement: Supplementary Figure 6 — KEGG enrichment of S1 vs S4 up-regulated genes. [file DataSheet_6.pdf]

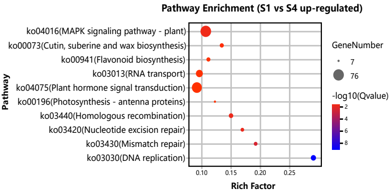
Pathway Enrichment (S1 vs S4 up-regulated)
Pathway
ko04016(MAPK signaling pathway - plant)
ko00073(Cutin, suberine and wax biosynthesis)
ko00941(Flavonoid biosynthesis)
ko03013(RNA transport)
ko04075(Plant hormone signal transduction)
ko00196(Photosynthesis - antenna proteins)
ko03440(Homologous recombination)
ko03420(Nucleotide excision repair)
ko03430(Mismatch repair)
ko03030(DNA replication)
0.10
0.15
0.20
0.25
Rich Factor
GeneNumber
7
76
-log10(Qvalue)
2
4
6
8
