# Supplementary material for: Time-series transcriptome provides insights into the gene regulation network involved in the icariin-flavonoid metabolism during the leaf development of Epimedium pubescens
Source: Front Plant Sci. 2023 Jun 12;14:1183481. doi: 10.3389/fpls.2023.1183481 (PMC10291196; doi:10.3389/fpls.2023.1183481)
Supplement: Supplementary file 12 [file Table_2.docx]

1. **Quality control of RNA-seq data**

**1.1 quality control using Trimmomatic**

java -jar /opt/biosoft/Trimmomatic-0.39/trimmomatic-0.39.jar PE -threads 100 /disk/70T/xcq/transcriptome/7_leaf_diff_development/data/R1.fastq /disk/70T/xcq/transcriptome/7_leaf_diff_development/data/R2.fastq R1.fastq R1.unpaired.fastq R2.fastq R2.unpaired.fastq ILLUMINACLIP:/opt/biosoft/Trimmomatic-0.39/adapters/TruSeq3-PE.fa:2:30:10 LEADING:3 TRAILING:3 SLIDINGWINDOW:4:15 MINLEN:36 TOPHRED33

1. **Reads alignment**

**2.1 Building an index of the reference genome**

hisat2-build -p 100 Ep.genome.fasta Ep.genome 1>hisat2-build.log 2>&1

- 1. **Reads alignment with HISAT2**

hisat2 -x /disk/70T/xcq/transcriptome/2_diff_tiss_892K/2.reads_alignment/hisat2/Ep.genome -p 14 --min-intronlen 20 --max-intronlen 20000 --dta --dta-cufflinks -1 $i.R1.fastq -2 $i.R2.fastq -S $i.sam --new-summary --summary-file $i.hisat2.summary

1. **Expression quantification**

**3.1 Remove reads that match multiple sites and have the same optimal match score**

perl -e 'while (<>) { unless (/^\@/) { @_ = split /\t/; next if \$_[4] < 10; } next if (/NH:i:(\d+)/ && \$1 > 1); print; }' $i > $x.sam; samtools sort -o $x.bam -O BAM $x.sam

**3.2 Expression of quantitative results calculation using R subread**

Rscript /disk/70T/xcq/transcriptome/1_Anth/3.quantification/script/run-featurecounts.R -b /disk/70T/xcq/transcriptome/7_leaf_diff_development/3.quantification/Rsubread/$i.bam -g /disk/70T/xcq/transcriptome/6_High_low_content/5_5/2.reads_alignment/hisat2/Ep_final.gtf -o $i

1. **Merge the results of expression of all samples**

ls /disk/70T/xcq/transcriptome/7_leaf_diff_development/3.quantification/Rsubread/*.count > genes.quant_files.txt

sudo perl script/abundance_estimates_to_matrix.pl --est_method featureCounts --quant_files genes.quant_files.txt --out_prefix genes

1. **PCA analysis of gene expression and metabolites content data**

R

library("FactoMineR")

library("factoextra")

res.pca <- prcomp(peak_area_2[, 4:12], scale. = TRUE)

pdf("pca_content.pdf")

fviz_pca_biplot(res.pca,

# Fill individuals by groups

label = "all",

pointshape = 21,

pointsize = 2.5,

fill.ind = peak_area_2$group1,

col.ind = "black",

# Color variable by groups

col.var = factor(c("Epimedin_A", "Epimedin_B", "Epimedin_C", "Icariin","quinovosyl_icariin","Anhydroicaritin_3_rha_oac_xyl_7_glc","Anhydroicaritin_3_rha_oac_xyl_oac_7_glc","Rha_icariside_2","Icariside_2")),addEllipses = TRUE, ellipse.type = "convex",

legend.title = list(fill = "group1", color = "Clusters"),

repel = TRUE # Avoid label overplotting

)+

ggpubr::fill_palette("jco")+ # Indiviual fill color

ggpubr::color_palette("npg") # Variable colors

dev.off()

####################################################################

1. **TO-GCNs**

#!bash

pwd=`pwd`

mkdir $pwd/4_togcn4

cd $pwd/4_togcn4

bash /disk/70T/xcq/transcriptome/7_leaf_diff_development/example/20220411_XCQ_TO_GCN/2_scripts/CutoffGcnMFS.sh -a /disk/70T/xcq/transcriptome/7_leaf_diff_development/example/20220411_XCQ_TO_GCN/1_data/allgenes.log2.tsv -b /disk/70T/xcq/transcriptome/7_leaf_diff_development/example/20220411_XCQ_TO_GCN/1_data/wd40TF_meta.tsv -i /disk/70T/xcq/transcriptome/7_leaf_diff_development/example/20220411_XCQ_TO_GCN/1_data/group.info

# GCN 15 15 ../cutoff/TF_cutoff.tsv ../cutoff/all_cutoff.tsv 0.85 0.85 -0.61 -0.61

# TO-GCN 15 15 /home/chaoqunxu/01_research/3_/4_togcn4/cutoff/all_cutoff.tsv /home/chaoqunxu/01_research/3_/4_togcn4/cutoff/all_cutoff.tsv 0.85 0.85 seed.txt 0

# cutoff

mkdir -p $pwd/4_togcn4/gcn_0.85

cd $pwd/4_togcn4/gcn_0.85

GCN 15 15 ../cutoff/TF_cutoff.tsv ../cutoff/all_cutoff.tsv 0.85 0.85 -0.61 -0.61

awk -F "," 'NR>1{print $1"\t"$3}' C1+C2+.csv | sort -u | awk '!(SEEN[$1,$2]++) && !(($2,$1) in SEEN)' > 0.85_gene.network

grep "others" ../wd40_attribute.tsv | awk 'BEGIN{OFS="\t";print "sourceid\ttargetid"}ARGIND==1{a[$1]=$1}ARGIND==2{if($2 in a) next;else print}' - 0.85_gene.network > 0.85_TF_gene.network

# togcn

mkdir -p $pwd/4_togcn4/togcn/Ebr04G048560_0.85

cd $pwd/4_togcn4/togcn/ Ebr04G048560_0.85

echo " Ebr04G048560" > seed.txt

TO-GCN 15 15 $pwd/4_togcn4/cutoff/all_cutoff.tsv $pwd/4_togcn4/cutoff/all_cutoff.tsv 0.85 0.85 seed.txt 0

awk -F "," 'NR>1{print $1"\n"$3}' $pwd/4_togcn4/gcn_0.85/C1+C2+.csv | sort -u | grep -Ff - TF_level.csv | awk -F "," '{if($2==1)gsub(/1/,"2",$2);print $1"\t"$2-1}' | sort -k 2n | awk 'BEGIN{print "geneid\tlevel"}{print $1"\tL"$2}' > row.info

awk '{print $1"\n"$2}' $pwd/4_togcn4/gcn_0.85/0.85_TF_gene.network | sort -u | awk 'ARGIND==1{a[$1]=$0}ARGIND==2{if($1 in a)print a[$1]}' $pwd/4_togcn4/wd40_attribute.tsv - | awk 'BEGIN{OFS="\t";print "geneid\tclass1\tclass2\tclass3\tTForNot\tlevel"}ARGIND==1{a[$1]=$0}ARGIND==2{if($1 in a)print a[$1]"\t"$2}' - row.info > Ebr04G048560_0.85.attribute.tsv

####################################################################

1. **WGCNA**

**7.1 check missing value and filter**

##filter

meanTPM=0.5 ####the threshold can be changed---过滤标准，可以修改

n=nrow(datExpr0)

datExpr0[n+1,]=apply(datExpr0[c(1:nrow(datExpr0)),],2,mean)

datExpr0=datExpr0[1:n,datExpr0[n+1,] > meanTPM]

dim(datExpr0)

#[1] 33 21358

filtered_TPM=t(datExpr0)

filtered_TPM=data.frame(rownames(filtered_TPM),filtered_TPM)

names(filtered_TPM)[1]="sample"

head(filtered_TPM)

write.table(filtered_TPM, file="WGCNA_TPM_filter.txt",row.names=F, col.names=T,quote=FALSE,sep="\t")

gsg = goodSamplesGenes(datExpr0, verbose = 3)

str(gsg)

gsg$allOK #如果返回是TRUE，无需进行这一步，返回是FALSE则要进行基因剔除

#datExpr = datExpr0[gsg$goodSamples, gsg$goodGenes]

datExpr = datExpr0

dim(datExpr)

#[1] 33 21358

**7.2 sample cluster**

sampleTree = hclust(dist(datExpr), method = "average")

# load trait data

read.table("/disk/70T/xcq/transcriptome/7_leaf_diff_development/9.WGCNA/WGCNA_inputdata/traits",header = T)

names(traitData)

allTraits <- traitData[,c(2:6)]

row.names(allTraits) <- traitData[,1]

dim(allTraits)

#[1] 33 5

names(allTraits)

# Form a data frame analogous to expression data that will hold the traits.

tpmSamples = rownames(datExpr)

traitSamples = rownames(allTraits)

traitRows = match(tpmSamples, traitSamples)

datTraits = allTraits[traitRows,]

dim(datTraits)

rownames(datTraits)

collectGarbage()

# Re-cluster samples

sampleTree2 = hclust(dist(datExpr), method = "average")

# Convert traits to a color representation: white means low, red means high, grey means missing entry

traitColors = numbers2colors(datTraits, signed = FALSE)

# Plot the sample dendrogram and the colors underneath.

#sizeGrWindow(12,12)

pdf(file="3_leaf_dendrogram_and_trait_heatmap.pdf",width=12,height=12)

plotDendroAndColors(sampleTree2, traitColors,

groupLabels = names(datTraits),

main = "Sample dendrogram and trait heatmap")

dev.off()

write.table(datTraits, file="WGCNA_datTraits.txt",row.names=T, col.names=T,quote=FALSE,sep="\t")

**7.3 Network construction**

**7.3.1 soft threshold determination**

powers = c(c(1:10), seq(from = 12, to=20, by=2))

# Call the network topology analysis function

sft = pickSoftThreshold(datExpr, powerVector = powers, verbose = 5)

# Plot the results:

pdf("4_softthreshold_allleaf_gene.pdf")

par(mfrow = c(1,2)) #op <- par(no.readonly = TRUE), and then:# par(op)

cex1 = 0.9

Scale-free topology fit(n/v) index as a function of the soft-thresholding power

plot(sft$fitIndices[,1], -sign(sft$fitIndices[,3])*sft$fitIndices[,2],

xlab="Soft Threshold (power)",ylab="Scale Free Topology Model Fit,signed R^2",

type="n",

main = paste("Scale independence"))

text(sft$fitIndices[,1], -sign(sft$fitIndices[,3])*sft$fitIndices[,2],

labels=powers,cex=cex1,col="red")

abline(h=0.90,col="red")

dev.off()

**7.3.2 One-step network construction and module detection. (note:change maxBlockSize = 5000 to 15000)**

net_power3 = blockwiseModules(datExpr, power = 12,

TOMType = "unsigned",

minModuleSize = 50,

reassignThreshold = 0,

mergeCutHeight = 0.3,

numericLabels = TRUE,

pamRespectsDendro = FALSE,

saveTOMs = TRUE,

saveTOMFileBase = "TOM_leaf_gene",

maxBlockSize = 25000,

verbose = 3)

**7.4. Relating modules to traits and identifying important genes**

**7.4.1 Quantifying module-trait associations**

# Define numbers of genes and samples

nGenes = ncol(datExpr)

nSamples = nrow(datExpr)

# Recalculate MEs with color labels

MEs0 = moduleEigengenes(datExpr, moduleColors)$eigengenes

MEs = orderMEs(MEs0)

moduleTraitCor = cor(MEs,datTraits,use = "p")

moduleTraitPvalue = corPvalueStudent(moduleTraitCor, 33)

# display correlations and their p-values

textMatrix = paste(signif(moduleTraitCor, 2), "\n(",

signif(moduleTraitPvalue,1), ")", sep = "")

dim(textMatrix) = dim(moduleTraitCor)

write.table(MEs, file="5_WGCNA_MEs.txt",row.names=T, col.names=T,quote=FALSE,sep="\t")

write.table(textMatrix, file="5_WGCNA_CorPvalueMatrix.txt",row.names=T, col.names=T,quote=FALSE,sep="\t")

write.table(moduleTraitCor, file="5_WGCNA_moduleTraitCor.txt",row.names=T, col.names=T,quote=FALSE,sep="\t")

write.table(moduleTraitPvalue, file="5_WGCNA_moduleTraitPvalue.txt",row.names=T, col.names=T,quote=FALSE,sep="\t")

# Display the correlation values within a heatmap plot with color legend, row and column annotation, and optional text within the heatmap.

pdf(file="6_M_trait_cor_leaf.pdf")

par(mar = c(6, 8, 3, 3))

labeledHeatmap(Matrix = moduleTraitCor,

xLabels = colnames(moduleTraitCor),

yLabels = names(MEs),

ySymbols = names(MEs),

colorLabels = FALSE,

colors = blueWhiteRed(50),

textMatrix = textMatrix,

setStdMargins = FALSE,

cex.text = 0.2,

cex.lab.x = 0.4,

zlim = c(-1,1),

main = paste("Module-trait relationships"))

dev.off()

###############################################################################
